# A systematic exploration of unexploited genes for oxidative stress in Parkinson’s disease

**DOI:** 10.1038/s41531-024-00776-1

**Published:** 2024-08-17

**Authors:** Takayuki Suzuki, Hidemasa Bono

**Affiliations:** 1 Graduate School of Integrated Sciences for Life, Hiroshima University, 3-10-23 Kagamiyama, Higashi-Hiroshima, Hiroshima 739-0046 Japan; 2 Genome Editing Innovation Center, Hiroshima University, 3-10-23 Kagamiyama, Higashi-Hiroshima, Hiroshima 739-0046 Japan; 3 Database Center for Life Science (DBCLS), Joint Support-Center for Data Science Research, Research Organization of Information and Systems (ROIS), 178-4-4 Wakashiba, Kashiwa, Chiba 277-0871 Japan

**Keywords:** Genetic databases, Genomics, Systems biology

## Abstract

Human disease-associated gene data are accessible through databases, including the Open Targets Platform, DisGeNET, miRTex, RNADisease, and PubChem. However, missing data entries in such databases are anticipated because of curational errors, biases, and text-mining failures. Additionally, the extensive research on human diseases has led to challenges in registering comprehensive data. The lack of essential data in databases hinders knowledge sharing and should be addressed. Therefore, we propose an analysis pipeline to explore missing entries of unexploited genes in the human disease-associated gene databases. Using this pipeline for genes in Parkinson’s disease with oxidative stress revealed two unexploited genes: nuclear protein 1 (*NUPR1)* and ubiquitin-like with PHD and ring finger domains 2 (*UHRF2)*. This methodology enhances the identification of underrepresented disease-associated genes, facilitating easier access to potential human disease-related functional genes. This study aims to identify unexploited genes for further research and does not include independent experimental validation.

## Introduction

Human disease research is a significant area in biology. For example, querying “parkinson disease” [All Fields] in the PubMed literature database yields 94,062 literatures (11 February 2024). Subsequently, research findings are curated by experts or extracted through text-mining methodologies to register in databases that facilitate collective intelligence. For instance, the Clinical Genome Resource (ClinGen)^1^, developed by the National Institutes of Health (NIH), curates, assesses, and disseminates aggregated genetic and disease associations as public data. The GWAS Catalog^2^ compiles genome-wide association data, including single-nucleotide polymorphisms (SNPs) with associated disease risks. Open Targets Platform^3^ integrates data from ClinGen and GWAS Catalog, and other public human disease-related databases, including CRISPRBrain^4^, Open Targets Genetics^5^, Gene Burden^6,7^, and Europe PMC^8,9^. Additional databases, including DisGeNET (expert-curated integrated gene-disease associations until March 2021)^10^, miRTex (specialized in text-mining approach-extracted microRNA)^11^, RNADisease (specialized in collecting non-coding RNAs with integrated approaches of curating and text-mining)^12^, and PubChem (specialized in extracted data from PubMed)^13^ offer complementary insights. They use various combinations of integration of data and specific text-mining methods and focus on RNA-disease links, capturing disease-gene associations that may not be included in the Open Targets Platform. These five databases offer comprehensive access to the latest human genes that have been implicated as functionally related to the disease.

As previously mentioned, PubMed database contained 94,062 studies related to PD. With these publications and accompanying data, missing data entries for disease-related functional genes are anticipated in databases^14^. Factors that contribute to missing disease-related genes may include challenges in computationally accessing biomedical statement contexts and supplementary data in the literature, oversights or biases by curators, and text-mining failures. These unexploited genes, which can be referred to as false-negative genes in current gene-disease association databases, represent incomplete dissemination of prior knowledge, potentially hindering research advancement and the development of disease prevention and treatment strategies. The manual identification of missing data entries across the literature databases requires substantial human resources and time expenditures. Therefore, we propose an approach to identify unexploited genes, which are missing data entries in five relevant databases. Based on our previous research on oxidative stress (OS)^15,16^, we selected PD, which exhibits pathological associations with OS, to demonstrate the efficacy of our methodology for identifying unexploited genes.

PD is a neurodegenerative disorder affecting over 6 million patients worldwide^17^. The primary symptoms observed in patients with PD include unilateral rigidity, bradykinesia, tremors, and non-motor symptoms, such as cognitive dysfunction^18–20^. The defining characteristics of PD include disordered α-synuclein aggregation, Lewy body formation, and significant loss of dopaminergic (DA) neurons in the substantia nigra, resulting in depleted dopamine levels, causing motor and cognitive deficits^18,20–23^. PD has been considered to be closely associated with the biological phenomenon, OS, wherein reactive oxygen species (ROS) and nucleophiles both contribute to and are generated by aggregating α-synuclein, Lewy bodies, and DA neuron loss^24^. Because OS is characterized by an imbalance between ROS levels and antioxidant defenses, substantial evidence has implicated it in PD pathogenesis^24^. As current therapies focus on symptomatic treatment, such as dopamine replacement, rather than root-cause therapy, understanding the molecular mechanisms of PD symptoms associated with OS is crucial for developing more effective therapies or biomarkers^25^. Querying “parkinson disease” [All Fields] AND “oxidative stress” [All Fields] in PubMed database yields 4061 publications (as of 15 February 2024), and manually reviewing all 4061 articles to sequentially identify unexploited genes would be labor-intensive. Therefore, the discovery of unexploited genes as candidates for the field of PD and OS research is necessary to further understand their underlying mechanisms.

To identify unexploited genes, we developed an analytical pipeline consisting of four significant stages. First, we curated a candidate gene set based on disease-relevant gene expression data. Second, we classified candidate genes based on the presence or absence of disease associations in the five applicable databases. Third, we refined the genes likely to be functional, leveraging data from transcriptome meta-analysis and transcriptome-wide association studies (TWAS). Finally, we manually searched the refined gene list to identify the unexploited genes with documented disease associations in the literature, but no links in the five databases. To identify genes related to OS in PD, we discovered two unexploited PD genes: nuclear protein 1 (*NUPR1)*, and ubiquitin-like with PHD and ring finger domains 2 (*UHRF2)*. The proposed approach and its findings will facilitate the identification of unexploited genes missing from databases, thereby advancing future research on human diseases.

## Results

### Overview of the pipeline [Fig. 1]

**Fig. 1:**
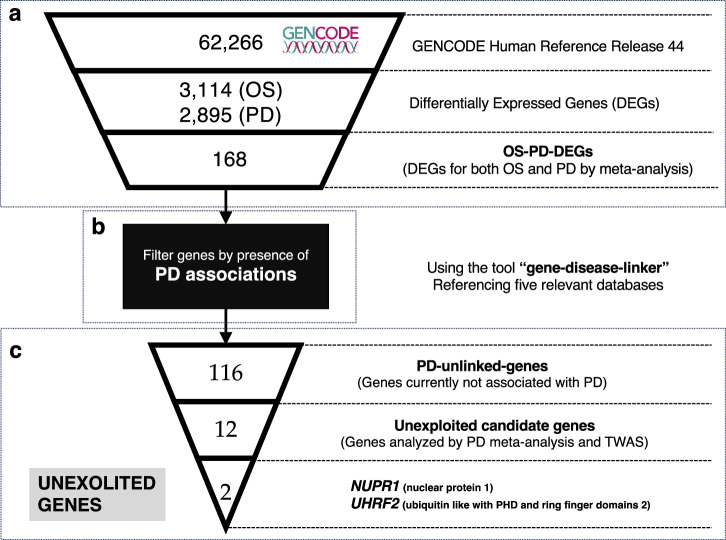
Overview of the pipeline filtering the number of candidates to search unexploited genes. **a** Transcriptome meta-analysis to retrieve differentially expressed genes (DEGs) in both oxidative stress (OS) and Parkinson’s disease (PD) (*n* = 168). **b** Gene-disease-linker (see Methods) filters the 168 candidate genes into PD-unlinked-genes based on gene-PD association with evidence studies. **c** PD-unlinked genes are further filtered using transcriptome-wide association studies (TWAS) and PD meta-analysis results to obtain unexploited candidate genes. A manual search of unexploited candidate genes revealed two unexploited genes.

Our stepwise methodology entailed the following: (1) Transcriptomic meta-analysis and literature mining were independently conducted to identify differentially expressed genes (DEGs) associated with OS and Parkinson’s disease (PD) in the human brain [Fig. 1a]. These outputs were compared to extract the dysregulated genes in both OS and PD (OS-PD-DEGs, *n* = 168) [Fig. 1a]. (2) The 168 candidate genes in OS-PD-DEGs were categorized into two subsets based on their associations with PD according to relevant databases (Open Targets Platform^3^, DisGeNET^10^, miRTex^11^, RNADisease^12^, and PubChem^13^). Each database collects information on the relationship between gene and disease in its unique way. Genes were categorized as “PD-linked-genes” if any database showed a connection to PD, and as “PD-unlinked-genes” if no databases showed such a connection. As a result, 116 genes were classified as PD-unlinked-genes [Fig. 1b], whereas the remaining 52 genes exhibited PD associations and classified as PD-linked-genes. (3) To identify genes with functions, we filtered PD-unlinked-genes using data from the PD transcriptome meta-analysis and TWAS, with 12 genes (unexploited candidate genes) remaining for the last step. 4) Finally, two unexploited genes *(NUPR1* and *UHRF2*) were discovered by manually searching PubMed Central for data on unexploited candidate genes [Fig. 1c]. The source data for identifying DEGs and the thresholds for the meta-analysis are potential confounders in this pipeline. However, to enhance the robustness and ensure the quality of identifying unexploited genes, we implemented two strategies: manual search and verification of unexploited genes at the end of the pipeline, and manual data curation for identifying candidate genes at the beginning of the pipeline.

### Collection of DEGs through OS

A transcriptomic meta-analysis was conducted on 122 paired RNA-sequencing (RNA-seq) datasets^26–33^ from cultured human cells related to the brain to identify DEGs associated with OS. Each pair comprised an OS sample and a matched normal condition sample from the same original study. Specifically, the transcriptomes of neurons, astrocytes, and neural progenitor cells [Supplementary Table 1]^34^ under OS or normal conditions were compiled from the Gene Expression Omnibus (GEO) database^35^. Oxidative stressors included radiation, hydrogen peroxide, rotenone, 1-methyl-4-phenylpyridinium (MPP^+^), paraquat, 6-hydroxydopamine (oxidopamine), methyl mercury chloride, and zinc [Table 1] [Supplementary Table 1]^34^. A total of 3114 genes (5% of all genes whose expression was quantified, termed OS-DEGs), were collected as DEGs, consisting of 1557 most upregulated and downregulated genes [Fig. 1a] with an ON_score of 1.5. Of the 357 genes in “GO:0006979: response to oxidative stress”, 37 possessed Ensembl IDs annotated to were included in OS-DEGs (*p* value: 2.671*e^−5^).

**Table 1:** Number of data pairs retrived for (a) each source of oxidative stress and (b) each type of cell culture

A	
Source of oxidative stress	Number of data pair
1-methyl-4-phenylpyridinium (MPP)	35
Rotenone	19
H_2_O_2_	17
Radiation	15
Paraquat	9
6-Hydroxydopamine	9
Methyl mercury Chloride	9
Zinc bis	9
Total	122

B	
Types of cultured cells	Number of data pair
Neuron	83
Astrocyte	24
Neural progenitor	15
Total	122

### Collection of DEGs through PD

**Fig. 2:**
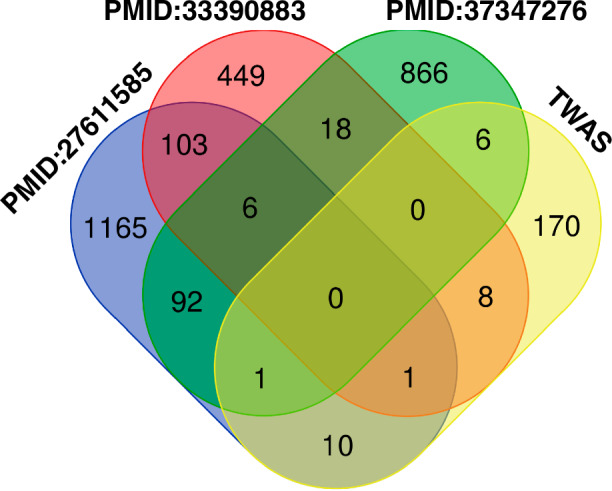
Venn diagram visualizing overlaps of PD-DEGs (*n* = 2895) among the studies. PMID represents PubMed ID. Transcriptome-wide association studies (TWAS) represents gene sets acquired from the TWAS-Atlas.

**Fig. 3:**
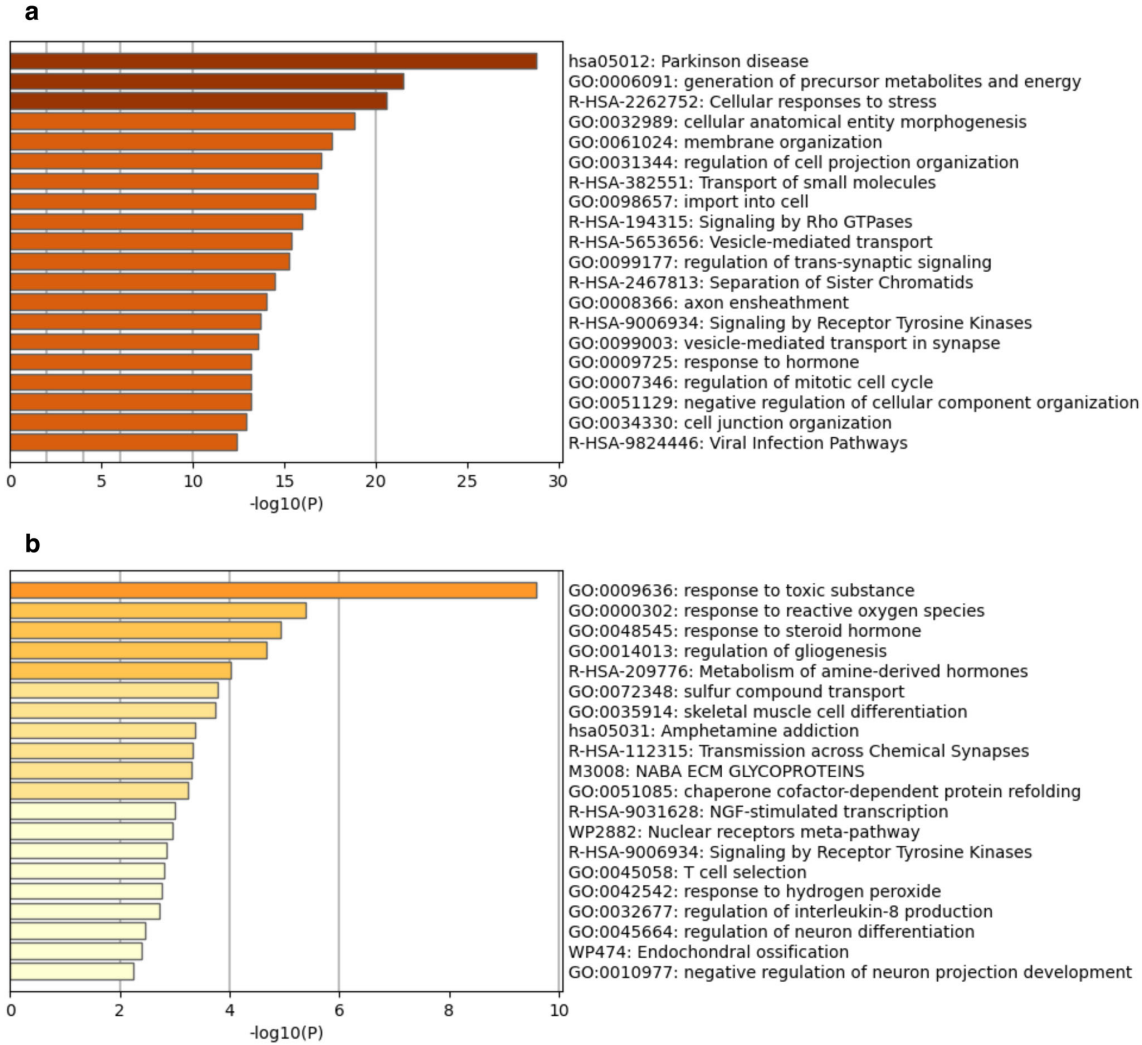
Results of gene set enrichment analysis (GSEA). **a** Results of GSEA of the PD-DEGs (*n* = 2763, unrecognized genes by Metascape, which are unstudied genes lacking NCBI gene IDs). **b** Results of GSEA of the differentially expressed genes (DEGs) commonly dysregulated in both oxidative stress (OS) and Parkinson’s disease (PD) (*n* = 168).

Because existing meta-analyses have delineated DEGs associated with PD, we curated and compiled PD-DEG sets from three relevant studies^36–38^ [Table 2]. These studies performed meta-analyses of PD transcriptomic data to derive robust gene expression signatures for the disease. Additionally, PD-associated genes identified through TWAS in the brain tissue were obtained from the TWAS-Atlas database^39^ and incorporated. In total, the PD-DEG comprised 2895 unique genes (1378 from PMID:27611585^36^, 585 from PMID: 33390883^37^, 989 from PMID: 37347276^38^, and 196 from the TWAS-Atlas) [Fig. 2]. Gene set enrichment analysis (GSEA) confirmed the signature of the PD-DEG compilation, with the most enriched term as “has05012: Parkinson disease” [Fig. 3a]. Collating PD-DEG lists from multiple large-scale omics studies and databases generated an extensive catalog of genes dysregulated in PD for subsequent analyses.

**Table 2:** Metadata for curated data for meta-analysis of Parkinson’s disease (PD)

PubMed ID	Article title	Published data	Data type	RNA source	Control	Research target (PD)	Control runs	PD runs	The number of DEGs	Method to analyze transcriptome
37347276	Transcriptomic profiling of Parkinson’s disease brains reveals disease stage specific gene expression changes	2023 June 22	RNA-seq	frontal cortex (the superior frontal gyrus at the level of anterior cornua of lateral ventricles corresponding to Brodmann area 8 or 9)	Braak stage 0	Braak stage 5 and 6	23	42	989	Salmon version 1.3.0 and DESeq2 version 1.34.0
27611585	Meta-analysis of Parkinson’s Disease Transcriptome Data Using TRAM Software: Whole Substantia Nigra Tissue and Single Dopamine Neuron Differential Gene Expression	2016 Sep 9	MicroArray	substantia nigra (post-mortem snap-frozen SN whole tissue) and dopaminergic neurons (from laser capture microdissection)	Non-PD patient	PD patient	130	151	1378	TRAM (Transcriptome Mapper) software version 1.2
33390883	Meta-analysis of differentially expressed genes in the substantia nigra in Parkinson’s disease supports Phenotype-specific Transcriptome Changes	2020 Dec 18	MicroArray	substantia nigra	Non-PD patient	PD patient	62	71	585	MetaMA version.3.1.2

### Collection of DEGs in both PD and OS

Comparative analysis of the 3114 OS-DEGs and 2895 Parkinson’s disease (PD-DEGs) revealed 168 genes dysregulated in both conditions (termed as OS-PD-DEGs) [Fig. 1a]. Of these, 132 were protein-coding, and 36 were non-coding RNA/pseudogene/small nucleolar RNA or unknown. GSEA of the 168-DEGs overlapping genes exhibited significant enrichment for “GO:0009636: response to toxic substance” and “GO:0000302: response to reactive oxygen species” [Fig. 3b], confirming their relevance to these biological processes. Mining of PD associations using the gene-disease-linker^40^ tool identified 52 OS-PD-DEGs with previously reported PD associations (PD-linked genes). The remaining 116 genes lacking known PD connections were termed PD-unlinked-genes [Fig. 1c]. The results of the gene-disease-linker using OS-PD-DEGs are listed in Supplementary Table 2^34^. The columns in Supplementary Table 2 are described as follows: ENSG: Ensemble gene ID, PD_log2(fold change)_PMID: Log_2_ fold change based on the original papers with PMID specified in the column name, TWAS: whether a gene was indicated in the TWAS, availability of associations: indicates if the gene exhibits association with PD (yes) or not (no), Evidence: databases providing evidence for an association between the given gene and the PD, NU_PMIDs_PD: Number of studies indicating an association between the given gene and PD, NU_PMIDs_NCBI: Number of studies associated with the given gene based on gene2pubmed^41^, PMIDs_PD: PubMed IDs for studies indicating an association between the given gene and PD, and PMIDs_NCBI: PubMed IDs for studies associated with the given gene based on gene2pubmed.

### Analysis of PD-unlinked-genes

Among the 116 PD-unlinked genes, six were identified with TWAS Z scores in the supplementary data of two studies with TWAS [Table 3, column: original TWAS]. Five of these genes were part of a set of 711 genes suggested to confer PD risk in the supplementary data of PMID: 33523105^42^. However, the main texts of the study (PMID: 33523105) lacked mention of these five genes. *MEI1* was one of the 44 genes implicated in dorsolateral prefrontal cortex PD associations in the TWAS from PMID: 30824768^43^, again lacking textual descriptions of *MEI1* within the study (PMID: 30824768). As these six genes were indicated to contribute to PD based on the TWAS results, we presumed them as suitable candidates for assessing the evidence or implications of their involvement in PD molecular mechanisms in the subsequent study mining step of our pipeline.

**Table 3:** Metadata for 12 unexploited candidate genes

Ensembl gene ID	GeneSymbol	ON score	PD_log2(fold change) (PMID:37347276)	PD log2(fold change) PMID:27611585	PD log2(fold_change) PMID:33390883	TWAS Z score	TWAS original paper (PMID)
ENSG00000099769	IGFALS	27	Not Found	Not Found	Not Found	4.45	33,523,105
ENSG00000125703	ATG4C	27	0.28	0.59	Not Found	Not Found	Not Found
ENSG00000112893	MAN2A1	26	0.49	0.58	Not Found	Not Found	Not Found
ENSG00000147854	UHRF2	25	0.29	0.58	Not Found	Not Found	Not Found
ENSG00000103932	RPAP1	24	Not Found	Not Found	Not Found	−5.04	33.523,105
ENSG00000167077	MEI1	24	Not Found	Not Found	Not Found	−3.37	30,824,768
ENSG00000267454	ZNF582−DT	23	Not Found	Not Found	Not Found	−3.61	33,523,105
ENSG00000176046	NUPR1	23	Not Found	0.73	0.61	4.36	33,523,105
ENSG00000184545	DUSP8	23	Not Found	Not Found	Not Found	4.20	33,523,105
ENSG00000164124	TMEM144	21	0.91	−0.83	Not Found	Not Found	Not Found
ENSG00000230487	PSMG3-AS1	−21	−0.38	−4.25	Not Found	Not Found	Not Found
ENSG00000080573	COL5A3	−30	−0.59	Not Found	0.50	Not Found	Not Found

Among the three PD gene expression meta-analyses examined, seven genes were suggested as PD-DEGs in more than two studies [Table 3, column: PD_log2(fold change)]. Notably, *NUPR1*, in addition to appearing in the supplementary data of the TWAS-based study, was also selected as a PD-DEG in two PD gene expression meta-analyses, indicating dysregulated expression of *NUPR1* in PD across three studies. Additionally, these seven genes were unexploited candidate genes in the subsequent step. A total of 12 genes [Table 3] have not been previously mentioned in their molecular association with PD as textual description in the main texts of studies; however, the sequence data indicated an association. These 12 genes were selected as unexploited candidate genes for the subsequent step, searching the full-text literature for molecular mechanistic evidence or hypotheses associated with them.

### Identification of unexploited genes

We searched the biomedical full-text literature database PubMed Central with the query “GENE_NAME[All Fields] AND parkinson[All Fields]” to look for unexploited genes (See Methods). Among the 12 genes examined, *NUPR1*, and *UHRF2* were identified as unexploited based on statements in the literature indicating their involvement in PD molecular mechanisms [Table 4]. For *NUPR1*, the PMCID: PMC10734959^44^ was used to determine the gene as unexploited. *NUPR1* was identified as one of the top five ferroptosis-related hub genes in PD by the methodology using random forest and support vector machine models. Additionally, the association between NUPR1 and alterations of the immune microenvironment of PD patients was indicated by a correlation analysis of NUPR1 and immune characteristics. It was mentioned that “The present study also suggests that *NUPR1* is involved in PD, is positively correlated with PD, and is most likely involved in PD pathogenic mechanisms through ferroptosis and OS”. For *UHRF2*, the PMCID: PMC9775085^45^ was used to determine the gene as unexploited. This review article integrates prior knowledge and proposes that UHRF2 dysregulation contributes to PD progression. It was specifically mentioned as follows; “Altogether, it could be assumed that the dysregulation of *CPNE8*, *CADPS2*, or *UHRF2* contributes to PD progression via ERK activation induced by the *LRRK2* G2019S mutation”. Table 4 provides the PubMed Central query dates by the author, query results, evidentiary publications (PubMed Central IDs), and quoted text supporting the unexploited status of each gene (evidence statements in the research paper).

**Table 4:** Results of searching unexploited genes

Ensembl gene ID	GeneSymbol	Unexploited genes (yes or no)	Evidence (PMCID)	Evidence statements in research paper	Search query in PMC	Search date	The number of publications yielded
ENSG00000125703	ATG4C	no	Not Found	Not Found	ATG4C[All Fields] AND parkinson[All Fields]	2024Feb12	67
ENSG00000099769	IGFALS	no	Not Found	Not Found	IGFALS[All Fields] AND parkinson[All Fields]	2024Feb12	13
ENSG00000112893	MAN2A1	no	Not Found	Not Found	MAN2A1[All Fields] AND parkinson[All Fields]	2024Feb12	15
ENSG00000147854	UHRF2	yes	PMC9775085	“The downregulation of UHRF2 gene expression can inhibit the function of the gene involved in cell growth, thus affecting the growth of mDA neurons. In common, UHRF2, CPNE8, and CADPS2 were related to the ERK signaling pathway activated by LRRK2 G2019S. According to a previous report, the LRRK2 G2019S mutation affects neurite shortening via ERK activity (Fig. 2) [1]. Altogether, it could be assumed that the dysregulation of CPNE8, CADPS2, or UHRF2 contributes to PD progression via ERK activation induced by the LRRK2 G2019S mutation.”	UHRF2[All Fields] AND parkinson[All Fields]	2024Feb12	22
ENSG00000103932	RPAP1	no	Not Found	Not Found	RPAP1[All Fields] AND parkinson[All Fields]	2024Feb12	4
ENSG00000167077	MEI1	no	Not Found	Not Found	MEI1[All Fields] AND parkinson[All Fields]	2024Feb12	12
ENSG00000176046	NUPR1	yes	PMC10734959	“The top 5 genes were extracted as FRHGs in Parkinson’s disease (CISD1, SIRT2, NUPR1, ADAM23 and NEDD4L)”, “The present study also suggests that NUPR1 is involved in PD, is positively correlated with PD, and is most likely involved in PD pathogenic mechanisms through ferroptosis and OS.”	NUPR1[All Fields] AND parkinson[All Fields]	2024Feb12	64
ENSG00000184545	DUSP8	no	Not Found	Not Found	DUSP8[All Fields] AND parkinson[All Fields]	2024Feb12	7
ENSG00000267454	ZNF582-DT	no	Not Found	Not Found	ZNF582-DT[All Fields] AND parkinson[All Fields]	2024Feb12	0
ENSG00000164124	TMEM144	no	Not Found	Not Found	TMEM144[All Fields] AND parkinson[All Fields]	2024Feb12	3
ENSG00000230487	PSMG3-AS1	no	Not Found	Not Found	PSMG3-AS1[All Fields] AND parkinson[All Fields]	2024Jun11	1
ENSG00000080573	COL5A3	no	Not Found	Not Found	COL5A3[All Fields] AND parkinson[All Fields]	2024Jun11	19

*NUPR1*, and *UHRF2* were identified

## Discussion

In this study, we developed a pipeline to identify unexploited genes, that correspond to false-negative genes for a given disease against five databases that provide gene-disease associations. Additionally, it was used to treat PD associated with OS. Through integrated analysis of curated datasets, including transcriptomics and transcriptome-wide association results, we filtered the 62,266 genes down to 168 genes (OS-PD-DEGs) that exhibited dysregulation of gene expression in both PD and OS contexts. We subsequently classified OS-PD-DEGs into PD-unlinked and PD-linked genes based on existing evidence of their involvement in PD. We have further narrowed down the PD-unlinked genes to 12 unexploited candidate genes. Following a manual search of these 12 genes, *NUPR1*, and *UHRF2* were identified as unexploited genes that were absent from the current gene-disease associations databases. These unexploited genes are described as functionally associated with PMC9775085 and PMC10734949, yet they are not captured in public gene-disease association databases. Although not the focus of this study due to not meeting the criteria to judge PD unexploited genes, several studies^46,47^ have reported dysregulated expression due to PD, which enhances the reliability of the association to PD. Thus, this pipeline effectively discovers such overlooked unexploited genes, aiding in the identification of experimental candidate genes.

Although it is challenging to conclusively determine the reasons for missing these entries from databases, three potential explanatory factors have been hypothesized as outlined in the Introduction. First, the databases may not have been recently updated. Two evidentiary publications on unexploited genes were recent (published in 2022 and 2023). Therefore, the absence of registrations may be possible without updates. Although the Open Targets Platform notes bi-monthly updates, the source database update frequencies vary, potentially explaining the omissions. Second, text-mining extraction failures are possible. For example, text-mining from the Europe PMC relies on co-occurrences at the sentence level with several filtering rules to reduce noise^9^. Their extraction methodology excludes articles other than research articles and filters out associations that appear only once in the body of an article but not in the article’s title or abstract. Therefore, the *UHRF2*-PD association was excluded from the Open Targets Platform because the review article PMC9775085 was filtered out by the extraction system. Third, certain databases rely on expert manual curation for new data entry, which may be pending or induce human errors or biases. Using our analytical pipeline to identify unexploited genes helps mitigate these database limitations.

Additionally, the two unexploited genes identified were associated with OS. NUPR1 acts as a key inhibitor of ferroptosis by regulating lipocalin-2 (LCN2) expression to reduce iron accumulation and subsequent oxidative damage^48^. For *UHRF2*, a gene set involved in ROS, UV response, and oxidative phosphorylation was induced in the retinal tissue of Uhrf2-deficient mice^49^. Among the 52 PD-linked genes, the majority have established associations with PD and OS (for instance, *SLC18A2*^50,51^, *TXNIP*^52^, *NEFL*^53,54^, *MPO*^55–58^, *LINC00938*^59,60^). Therefore, the 168 OS-PD-DEGs are suggested to include not only well-known OS in PD research candidates, but also novel candidates.

Moreover, PD-linked genes with limited evidentiary publications may harbor false positives (genes that are flagged as disease-associated in a database containing evidence with inappropriate evidence). For example, we identified superoxide dismutase 3 (*SOD3)* as a false-positive result. *SOD3* was linked to PD through the Open Targets Platform with a single literature annotation, which, on closer inspection turns out that the contents in the literature claim the opposite, that no significant SNP-PD risk association are found for *SOD*3^61^. This example demonstrates that our pipeline has the potential to reveal false positive entries in the database.

Finally, we outline four limitations of the analysis pipeline used in this study. First, several pipeline steps require time-consuming manual efforts. The curation of RNA-seq data is required for gene expression analysis in the first step. Following the refinement of the unexploited candidate genes, PMC was manually searched to assess the status of each gene. These manual steps render the methodology unsuitable for the comprehensive identification of numerous unexploited genes. The second limitation is the potential error of extracting false-positive genes, which may appear disease-associated but have weak actual relevance. This is particularly possible in RNAdisease and DisGeNET, where genes are searched based on threshold scores indicating association strength. However, such false-positive genes can be manually verified by reviewing the associated supporting literature as outlined in the Discussion. Furthermore, since the aim of this study is to identify false negatives, false-positive genes are not expected to hinder this objective and, therefore, are not considered a significant issue. The third limitation is the potential for confounders to prevent the identification of unexploited genes. In this study, we selected the candidate gene sets, OS-PD-DEGs, from various meta-analysis results. Changing potential confounders, such as the type and quantity of RNA-seq data selected or the thresholds used in the meta-analysis, could alter the OS-PD-DEGs and, subsequently, the unexploited genes. Therefore, when using this pipeline, it is necessary to curate as many suitable samples as possible and test various thresholds. Lastly, the referenced gene-disease databases are not static but will evolve with database research progress. We selected five databases to maximize the disease-gene coverage presently. However, novel databases are likely to emerge and be integrated over time. Therefore, appropriate database selection based on contemporary availability is necessary.

## Methods

### Curation of public data for OS

To collect OS RNA-sequencing data, relevant datasets were manually curated from the GEO^35^ repository based on five criteria: (1) total RNA or polyA-enriched sequencing, (2) samples under conditions related to the definition of OS, (3) samples under conditions related to increased ROS levels, (4) availability of paired normal-state samples as a control, and (5) cell cultures with brain relevance (neurons, astrocytes, and progenitors). This resulted in 122 matched OS-normal sample pairs from 10 research groups for analysis as a result of curating started from August 2023 to 31 October 2023. Comprehensive details of the public datasets used in this study are listed in Supplementary Table 1^34^.

### Curation of public data for PD

To compile DEGs in PD, we conducted a manual survey searching the PubMed database. Meta-analyses of three published studies were found, and we extracted all reported DEGs. Additionally, genes queried for PD in the TWAS-Atlas database^39^ were incorporated into the PD-DEG list for downstream analysis. This curation was conducted from August 2023 to 31 October 2023. Comprehensive details regarding all the public datasets used in this study are listed in Table 2.

### OS meta-analysis

For RNA-seq data retrieval, processing, and quantification, we used Ikra^62^, an automated pipeline program for RNA-seq data of *Homo sapiens* and *Mus musculus*. The following pipeline comprised fasterq-dump (version.3.0.1)^63^, trim-galore (version.0.6.7)^64^, and salmon (version.1.4.0)^65^ processes, with reference transcript sets in GENCODE Release 44 (GRCh38.p14). The transcript IDs were converted into the gene IDs using tximport (http://bioconductor.org/packages/tximport/) [Supplementary Table 2]^34^. To retrieve DEGs across 122 paired RNA-seq, we devised an oxidative stress-normal-state score (ON-score) based on these datasets. Initially, the ON-ratio was calculated for each gene, representing the expression ratio between OS and normal states across all sample pairs (Eq. 1). Subsequently, genes were then categorized as upregulated, downregulated, or unchanged based on the ON-ratio exceeding a ±1.5-fold threshold. Furthermore, the ON-score for each gene was calculated using Eq. 2, which involved subtracting the number of downregulated samples from the number of upregulated samples. This scoring methodology was detailed extensively in a previous study^15^. The ON score measured how many of the 122 pairs of samples dysregulate the expression of each gene.

1 $${ON\; ratio}=\frac{{T}_{{OS}}+1}{{T}_{{Normal\; state}}+1}$$

2 $${ON\; score}={{Count\; number}}_{{upregulated}}-{{Count\; number}}_{{downregulated}}$$

### Comparison of OS meta-analysis method with DESeq2

To assess the statistical validity of the meta-analysis method, we compared its results with those obtained using DESeq2 (package version:1.44.0), a widely used tool in bioinformatics research. Setting the threshold at log2FoldChange ≧ |1| and a *p* value adjusted by the false discovery rate <0.05, 352 genes were identified. The overlap between the 3114 genes identified by meta-analysis and the 352 genes identified by DESeq2 was 89. We conducted Fisher’s exact test to determine whether there is a statistically significant correlation between the gene sets obtained by meta-analysis and DESeq2. The sample size of genes was 62,266, with 3114 genes from the meta-analysis, 352 genes from DESeq2, and an overlap of 89 genes. The calculation yielded a *p* value of 1.52e-37 at a significant level of 0.05. This indicates that the probability of such an overlap occurring by chance is extremely low, demonstrating a statistically significant correlation between the gene sets obtained by the meta-analysis and DESeq2 methods. The DESeq2 output [Supplementary Table 4], the list of 352 genes [Supplementary Table 4], the list of overlapped 89 genes [Supplementary Table 4], and the script for calculating the fisher’s exact test [Supplementary Data 5] are available in the Figshare repository^34^.

### Classifying genes by the availability of associations with PD

**Fig. 4:**
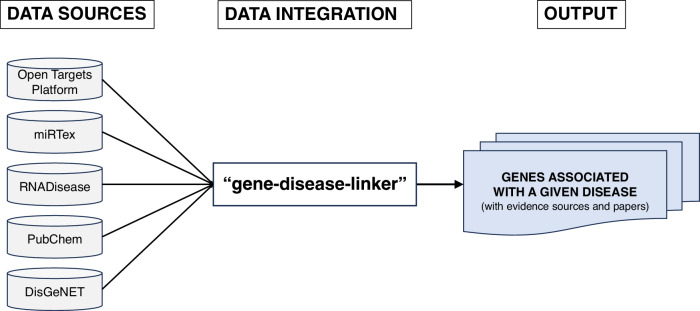
Overview of gene-disease-linker collecting information about gene-disease associations based on the relevant five databases.

To classify genes based on prior evidence linking them to a disease, we originally developed the tool called gene-disease-linker^40^ [Fig. 4]. The basic functionality of gene-disease-linker is to efficiently search five public gene-disease association databases, perform ID conversions, and organize search results. Using this tool, genes can be efficiently categorized as “linked” or “unlinked” for a specific disease by searching five public databases (accessed on 8 February 2024)—Open Targets Platform^3^, RNAdisease^12^, miRTex^11^, DisGeNET^10^, and PubChem^13^ - for gene-disease relationships. As this categorization relies on these five databases, it is crucial that they comprehensively cover disease-gene associations as much as possible. We investigated as many public databases as possible from August 2023 to February 2024, requiring each gene-disease association to include supporting references. As a result, we selected these five public databases.

The detailed description of gene-disease-linker is as follows: By inputting a text file of genes list and configuration file into gene-disease-linker, it outputs the search results from these five databases, thereby enabling the determination of whether there is a gene-disease association for each gene in the list regarding a specific disease. In cases a gene exhibiting an association with the disease, it also concurrently outputs the supporting literature with PubMed ID. In contrast, no gene-disease association is indicated if there is no supporting literature annotated to a gene. In this study, a gene-disease-linker was used to classify genes as either PD-linked (existing literature linking the gene to PD) or PD-unlinked (no evidence found) (executed on 8 February 2024). The source codes and usage of gene-disease-linker are available in the GitHub repository. The text file of the genes list and configuration file we used in this study are available in the GitHub repository (168genes.txt and config.yml, respectively). Also, all intermediate and output files from running the gene-disease-linker in this study are available in the results folder on GitHub.

### Criteria to judge PD unexploited genes

We searched gene names in PubMed Central, a full-text literature database, with the following query: “GENE_NAME[All Fields] AND Parkinson[All Fields]”. Within the retrieved articles, we analyzed the surrounding textual context of gene mentions to identify descriptions indicating or suggesting molecular functional relationships with PD. Only genes with mechanistic evidence or relationships reported in the literature were judged unexploited. Genes only listed among the DEGs without any statements related to functional implications were excluded from the unexploited.

### Other analysis

GSEA was performed using the web-based tool Metascape^66^. Shared genes among the various gene sets were visualized using a publicly available web-based Venn diagram generator (https://bioinformatics.psb.ugent.be/webtools/Venn/).

### Supplementary information

Supplemental Tables & Data

## Data Availability

The datasets curated, generated, and analyzed during this study are available in the figshare repository^34^.

## Code availability

The underlying code for the current study is available at gene-disease-linker^40^.
