# Supplementary material for: A systematic exploration of unexploited genes for oxidative stress in Parkinson’s disease
Source: NPJ Parkinsons Dis. 2024 Aug 17;10:160. doi: 10.1038/s41531-024-00776-1 (PMC11330442; doi:10.1038/s41531-024-00776-1)
Supplement: Supplementary file 1 — Supplemental Tables & Data [file 41531_2024_776_MOESM1_ESM.pdf]

**Supplementary Table1: Metadata for RNA-seq data from coltured human cells related to brain used for meta-analysis in oxidative stress.**

| SRA Project ID | Oxidative Stress | Control | Source of Stress (Reagent) | Concentration | cell type | Time | Library type | extracted molecule | Note |
|---|---|---|---|---|---|---|---|---|---|
| SRP239545 | SRR10832030 | SRR10832029 | H2O2 | 800microM | neuron (primary fibroblasts fron | 17 hours | PE | total RNA | |
| SRP239545 | SRR10832031 | SRR10832032 | H2O2 | 800microM | neuron (primary fibroblasts fron | 17 hours | PE | total RNA | |
| SRP239545 | SRR10832035 | SRR10832033 | H2O2 | 800microM | neuron (primary fibroblasts fron | 17 hours | PE | total RNA | |
| SRP239545 | SRR10832036 | SRR10832034 | H2O2 | 800microM | neuron (primary fibroblasts fron | 17 hours | PE | total RNA | |
| SRP260342 | SRR11719885 | SRR11719939 | paraquat | 5microM | neuron (LUHMES) | 6 hours | PE | polyA RNA | |
| SRP260342 | SRR11719886 | SRR11719940 | paraquat | 5microM | neuron (LUHMES) | 6 hours | PE | polyA RNA | |
| SRP260342 | SRR11719887 | SRR11719941 | paraquat | 5microM | neuron (LUHMES) | 6 hours | PE | polyA RNA | |
| SRP260342 | SRR11719888 | SRR11719942 | paraquat | 5microM | neuron (LUHMES) | 6 hours | PE | polyA RNA | |
| SRP260342 | SRR11719889 | SRR11719943 | paraquat | 5microM | neuron (LUHMES) | 6 hours | PE | polyA RNA | |
| SRP260342 | SRR11719890 | SRR11719944 | paraquat | 5microM | neuron (LUHMES) | 6 hours | PE | polyA RNA | |
| SRP260342 | SRR11719891 | SRR11719945 | paraquat | 5microM | neuron (LUHMES) | 6 hours | PE | polyA RNA | |
| SRP260342 | SRR11719892 | SRR11719946 | paraquat | 5microM | neuron (LUHMES) | 6 hours | PE | polyA RNA | |
| SRP260342 | SRR11719893 | SRR11719947 | paraquat | 5microM | neuron (LUHMES) | 6 hours | PE | polyA RNA | |
| SRP260342 | SRR11719894 | SRR11719939 | 1-methyl-4-phenylpyridinium (MPP) | 5microM | neuron (LUHMES) | 6 hours | PE | polyA RNA | |
| SRP260342 | SRR11719895 | SRR11719940 | 1-methyl-4-phenylpyridinium (MPP) | 5microM | neuron (LUHMES) | 6 hours | PE | polyA RNA | |
| SRP260342 | SRR11719896 | SRR11719941 | 1-methyl-4-phenylpyridinium (MPP) | 5microM | neuron (LUHMES) | 6 hours | PE | polyA RNA | |
| SRP260342 | SRR11719897 | SRR11719942 | 1-methyl-4-phenylpyridinium (MPP) | 5microM | neuron (LUHMES) | 6 hours | PE | polyA RNA | |
| SRP260342 | SRR11719898 | SRR11719943 | 1-methyl-4-phenylpyridinium (MPP) | 5microM | neuron (LUHMES) | 6 hours | PE | polyA RNA | |
| SRP260342 | SRR11719899 | SRR11719944 | 1-methyl-4-phenylpyridinium (MPP) | 5microM | neuron (LUHMES) | 6 hours | PE | polyA RNA | |
| SRP260342 | SRR11719900 | SRR11719945 | 1-methyl-4-phenylpyridinium (MPP) | 5microM | neuron (LUHMES) | 6 hours | PE | polyA RNA | |
| SRP260342 | SRR11719901 | SRR11719946 | 1-methyl-4-phenylpyridinium (MPP) | 5microM | neuron (LUHMES) | 6 hours | PE | polyA RNA | |
| SRP260342 | SRR11719902 | SRR11719947 | 1-methyl-4-phenylpyridinium (MPP) | 5microM | neuron (LUHMES) | 6 hours | PE | polyA RNA | |
| SRP260342 | SRR11719903 | SRR11719939 | Zinc dimethyldithiocarbamate | 2microM | neuron (LUHMES) | 6 hours | PE | polyA RNA | |
| SRP260342 | SRR11719904 | SRR11719940 | Zinc dimethyldithiocarbamate | 2microM | neuron (LUHMES) | 6 hours | PE | polyA RNA | |
| SRP260342 | SRR11719905 | SRR11719941 | Zinc dimethyldithiocarbamate | 2microM | neuron (LUHMES) | 6 hours | PE | polyA RNA | |
| SRP260342 | SRR11719906 | SRR11719942 | Zinc dimethyldithiocarbamate | 2microM | neuron (LUHMES) | 6 hours | PE | polyA RNA | |
| SRP260342 | SRR11719907 | SRR11719943 | Zinc dimethyldithiocarbamate | 2microM | neuron (LUHMES) | 6 hours | PE | polyA RNA | |
| SRP260342 | SRR11719908 | SRR11719944 | Zinc dimethyldithiocarbamate | 2microM | neuron (LUHMES) | 6 hours | PE | polyA RNA | |
| SRP260342 | SRR11719909 | SRR11719945 | Zinc dimethyldithiocarbamate | 2microM | neuron (LUHMES) | 6 hours | PE | polyA RNA | |
| SRP260342 | SRR11719910 | SRR11719946 | Zinc dimethyldithiocarbamate | 2microM | neuron (LUHMES) | 6 hours | PE | polyA RNA | |
| SRP260342 | SRR11719911 | SRR11719947 | Zinc dimethyldithiocarbamate | 2microM | neuron (LUHMES) | 6 hours | PE | polyA RNA | |
| SRP260342 | SRR11719912 | SRR11719939 | rotenone | 1microM | neuron (LUHMES) | 6 hours | PE | polyA RNA | |
| SRP260342 | SRR11719913 | SRR11719940 | rotenone | 1microM | neuron (LUHMES) | 6 hours | PE | polyA RNA | |
| SRP260342 | SRR11719914 | SRR11719941 | rotenone | 1microM | neuron (LUHMES) | 6 hours | PE | polyA RNA | |
| SRP260342 | SRR11719915 | SRR11719942 | rotenone | 1microM | neuron (LUHMES) | 6 hours | PE | polyA RNA | |
| SRP260342 | SRR11719916 | SRR11719943 | rotenone | 1microM | neuron (LUHMES) | 6 hours | PE | polyA RNA | |
| SRP260342 | SRR11719917 | SRR11719944 | rotenone | 1microM | neuron (LUHMES) | 6 hours | PE | polyA RNA | |
| SRP260342 | SRR11719918 | SRR11719945 | rotenone | 1microM | neuron (LUHMES) | 6 hours | PE | polyA RNA | |
| SRP260342 | SRR11719919 | SRR11719946 | rotenone | 1microM | neuron (LUHMES) | 6 hours | PE | polyA RNA | |
| SRP260342 | SRR11719920 | SRR11719947 | rotenone | 1microM | neuron (LUHMES) | 6 hours | PE | polyA RNA | |

| | | | | | | | | | |
|---|---|---|---|---|---|---|---|---|---|
| SRP260342 | SRR11719921 | SRR11719939 | 6-Hyroxydopamine | 1microM | neuron (LUHMES) | 6 hours | PE | polyA RNA | |
| SRP260342 | SRR11719922 | SRR11719940 | 6-Hyroxydopamine | 1microM | neuron (LUHMES) | 6 hours | PE | polyA RNA | |
| SRP260342 | SRR11719923 | SRR11719941 | 6-Hyroxydopamine | 1microM | neuron (LUHMES) | 6 hours | PE | polyA RNA | |
| SRP260342 | SRR11719924 | SRR11719942 | 6-Hyroxydopamine | 1microM | neuron (LUHMES) | 6 hours | PE | polyA RNA | |
| SRP260342 | SRR11719925 | SRR11719943 | 6-Hyroxydopamine | 1microM | neuron (LUHMES) | 6 hours | PE | polyA RNA | |
| SRP260342 | SRR11719926 | SRR11719944 | 6-Hyroxydopamine | 1microM | neuron (LUHMES) | 6 hours | PE | polyA RNA | |
| SRP260342 | SRR11719927 | SRR11719945 | 6-Hyroxydopamine | 1microM | neuron (LUHMES) | 6 hours | PE | polyA RNA | |
| SRP260342 | SRR11719928 | SRR11719946 | 6-Hyroxydopamine | 1microM | neuron (LUHMES) | 6 hours | PE | polyA RNA | |
| SRP260342 | SRR11719929 | SRR11719947 | 6-Hyroxydopamine | 1microM | neuron (LUHMES) | 6 hours | PE | polyA RNA | |
| SRP260342 | SRR11719930 | SRR11719939 | Methylmercury Chloride | 0.5microM | neuron (LUHMES) | 6 hours | PE | polyA RNA | |
| SRP260342 | SRR11719931 | SRR11719940 | Methylmercury Chloride | 0.5microM | neuron (LUHMES) | 6 hours | PE | polyA RNA | |
| SRP260342 | SRR11719932 | SRR11719941 | Methylmercury Chloride | 0.5microM | neuron (LUHMES) | 6 hours | PE | polyA RNA | |
| SRP260342 | SRR11719933 | SRR11719942 | Methylmercury Chloride | 0.5microM | neuron (LUHMES) | 6 hours | PE | polyA RNA | |
| SRP260342 | SRR11719934 | SRR11719943 | Methylmercury Chloride | 0.5microM | neuron (LUHMES) | 6 hours | PE | polyA RNA | |
| SRP260342 | SRR11719935 | SRR11719944 | Methylmercury Chloride | 0.5microM | neuron (LUHMES) | 6 hours | PE | polyA RNA | |
| SRP260342 | SRR11719936 | SRR11719945 | Methylmercury Chloride | 0.5microM | neuron (LUHMES) | 6 hours | PE | polyA RNA | |
| SRP260342 | SRR11719937 | SRR11719946 | Methylmercury Chloride | 0.5microM | neuron (LUHMES) | 6 hours | PE | polyA RNA | |
| SRP260342 | SRR11719938 | SRR11719947 | Methylmercury Chloride | 0.5microM | neuron (LUHMES) | 6 hours | PE | polyA RNA | |
| SRP139943 | SRR7000803 | SRR7000802 | radiation | 5Gy | iPS cell derived neural progenitor cell | | PE | total RNA | |
| SRP043644 | SRR1485146 | SRR1485145 | H2O2 | 200microM | primary astrocytes | 2 hours | SE | total RNA | RNA extracted 6 days after the initiation of treatment |
| SRP043644 | SRR1485147 | SRR1485144 | H2O2 | 200microM | primary astrocytes | 2 hours | SE | total RNA | RNA extracted 6 days after the initiation of treatment |
| SRP186270 | SRR8591329 | SRR8591320 | rotenone | 900nM | cerebellar astrocytes | 16 hours | SE | total RNA | |
| SRP186270 | SRR8591330 | SRR8591321 | rotenone | 900nM | cerebellar astrocytes | 16 hours | SE | total RNA | |
| SRP186270 | SRR8591331 | SRR8591322 | rotenone | 900nM | cerebellar astrocytes | 16 hours | SE | total RNA | |
| SRP186270 | SRR8591332 | SRR8591323 | rotenone | 900nM | cerebellar astrocytes | 16 hours | SE | total RNA | |
| SRP186270 | SRR8591333 | SRR8591324 | rotenone | 900nM | cerebellar astrocytes | 16 hours | SE | total RNA | |
| SRP186270 | SRR8591334 | SRR8591325 | rotenone | 900nM | cerebellar astrocytes | 16 hours | SE | total RNA | |
| SRP186270 | SRR8591335 | SRR8591326 | rotenone | 900nM | cerebellar astrocytes | 16 hours | SE | total RNA | |
| SRP186270 | SRR8591336 | SRR8591327 | rotenone | 900nM | cerebellar astrocytes | 16 hours | SE | total RNA | |
| SRP186270 | SRR8591337 | SRR8591328 | rotenone | 900nM | cerebellar astrocytes | 16 hours | SE | total RNA | |
| SRP186270 | SRR8591338 | SRR8591320 | rotenone | 900nM | cerebellar astrocytes | 16 hours | SE | total RNA | |
| SRP186270 | SRR8591339 | SRR8591321 | H2O2 | 150microM | cerebellar astrocytes | 2 hours | SE | total RNA | |
| SRP186270 | SRR8591340 | SRR8591322 | H2O2 | 150microM | cerebellar astrocytes | 2 hours | SE | total RNA | |
| SRP186270 | SRR8591341 | SRR8591323 | H2O2 | 150microM | cerebellar astrocytes | 2 hours | SE | total RNA | |
| SRP186270 | SRR8591342 | SRR8591324 | H2O2 | 150microM | cerebellar astrocytes | 2 hours | SE | total RNA | |
| SRP186270 | SRR8591343 | SRR8591325 | H2O2 | 150microM | cerebellar astrocytes | 2 hours | SE | total RNA | |
| SRP186270 | SRR8591344 | SRR8591326 | H2O2 | 150microM | cerebellar astrocytes | 2 hours | SE | total RNA | |
| SRP186270 | SRR8591345 | SRR8591327 | H2O2 | 150microM | cerebellar astrocytes | 2 hours | SE | total RNA | |
| SRP186270 | SRR8591346 | SRR8591328 | H2O2 | 150microM | cerebellar astrocytes | 2 hours | SE | total RNA | |
| SRP376418 | SRR19346970 | SRR19346978 | 1-methyl-4-phenylpyridinium (MPP) | 1mM | SH-SY5Y cells differentiated int | 48 hours | PE | total RNA | cancer derived cell line, 3D culture |
| SRP376418 | SRR19346971 | SRR19346979 | 1-methyl-4-phenylpyridinium (MPP) | 1mM | SH-SY5Y cells differentiated int | 48 hours | PE | total RNA | cancer derived cell line, 3D culture |
| SRP376418 | SRR19346972 | SRR19346980 | 1-methyl-4-phenylpyridinium (MPP) | 1mM | SH-SY5Y cells differentiated int | 48 hours | PE | total RNA | cancer derived cell line, 3D culture |

| | | | | | | | | | |
|---|---|---|---|---|---|---|---|---|---|
| SRP376418 | SRR19346973 | SRR19346981 | 1-methyl-4-phenylpyridinium (MPP) | 1mM | SH-SY5Y cells differentiated int | 48 hours | PE | total RNA | cancer derived cell line, 3D culture |
| SRP376418 | SRR19346974 | SRR19346982 | 1-methyl-4-phenylpyridinium (MPP) | 1mM | SH-SY5Y cells differentiated int | 48 hours | PE | total RNA | cancer derived cell line, 2D culture |
| SRP376418 | SRR19346975 | SRR19346983 | 1-methyl-4-phenylpyridinium (MPP) | 1mM | SH-SY5Y cells differentiated int | 48 hours | PE | total RNA | cancer derived cell line, 2D culture |
| SRP376418 | SRR19346976 | SRR19346984 | 1-methyl-4-phenylpyridinium (MPP) | 1mM | SH-SY5Y cells differentiated int | 48 hours | PE | total RNA | cancer derived cell line, 2D culture |
| SRP376418 | SRR19346977 | SRR19346985 | 1-methyl-4-phenylpyridinium (MPP) | 1mM | SH-SY5Y cells differentiated int | 48 hours | PE | total RNA | cancer derived cell line, 2D culture |
| SRP222829 | SRR10156158 | SRR10156149 | radiation | 15mGy | hESC-derived neural progenitor cells | | PE | total RNA | ES differentiate day 10 |
| SRP222829 | SRR10156159 | SRR10156150 | radiation | 15mGy | hESC-derived neural progenitor cells | | PE | total RNA | ES differentiate day 10 |
| SRP222829 | SRR10156160 | SRR10156151 | radiation | 15mGy | hESC-derived neural progenitor cells | | PE | total RNA | ES differentiate day 10 |
| SRP222829 | SRR10156161 | SRR10156152 | radiation | 15mGy | hESC-derived neural progenitor cells | | PE | total RNA | ES differentiate day 6 |
| SRP222829 | SRR10156162 | SRR10156153 | radiation | 15mGy | hESC-derived neural progenitor cells | | PE | total RNA | ES differentiate day 6 |
| SRP222829 | SRR10156166 | SRR10156149 | radiation | 500mGy | hESC-derived neural progenitor cells | | PE | total RNA | ES differentiate day 10 |
| SRP222829 | SRR10156167 | SRR10156150 | radiation | 500mGy | hESC-derived neural progenitor cells | | PE | total RNA | ES differentiate day 10 |
| SRP222829 | SRR10156168 | SRR10156151 | radiation | 500mGy | hESC-derived neural progenitor cells | | PE | total RNA | ES differentiate day 10 |
| SRP222829 | SRR10156169 | SRR10156152 | radiation | 500mGy | hESC-derived neural progenitor cells | | PE | total RNA | ES differentiate day 6 |
| SRP222829 | SRR10156170 | SRR10156153 | radiation | 500mGy | hESC-derived neural progenitor cells | | PE | total RNA | ES differentiate day 6 |
| SRP222829 | SRR10156171 | SRR10156154 | radiation | 500mGy | hESC-derived neural progenitor cells | | PE | total RNA | ES differentiate day 6 |
| SRP340073 | SRR16203693 | SRR16203686 | H2O2 | 100microM | Human fetal neural stem cells i: | 4 hours | PE | total RNA | |
| SRP340073 | SRR16203694 | SRR16203687 | H2O2 | 100microM | Human fetal neural stem cells i: | 4 hours | PE | total RNA | |
| SRP340073 | SRR16203695 | SRR16203688 | H2O2 | 100microM | Human fetal neural stem cells i: | 4 hours | PE | total RNA | |
| SRP358552 | SRR17898575 | SRR17898578 | 1-methyl-4-phenylpyridinium (MPP) | 100microM | iPSC-derived dopaminergic ne | 24 hours | PE | total RNA | |
| SRP358552 | SRR17898576 | SRR17898579 | 1-methyl-4-phenylpyridinium (MPP) | 100microM | iPSC-derived dopaminergic ne | 24 hours | PE | total RNA | |
| SRP358552 | SRR17898577 | SRR17898580 | 1-methyl-4-phenylpyridinium (MPP) | 100microM | iPSC-derived dopaminergic ne | 24 hours | PE | total RNA | |
| SRP358552 | SRR17898587 | SRR17898590 | 1-methyl-4-phenylpyridinium (MPP) | 100microM | iPSC-derived dopaminergic ne | 24 hours | PE | total RNA | |
| SRP358552 | SRR17898588 | SRR17898591 | 1-methyl-4-phenylpyridinium (MPP) | 100microM | iPSC-derived dopaminergic ne | 24 hours | PE | total RNA | |
| SRP358552 | SRR17898589 | SRR17898592 | 1-methyl-4-phenylpyridinium (MPP) | 100microM | iPSC-derived dopaminergic ne | 24 hours | PE | total RNA | |
| SRP358552 | SRR17898584 | SRR17898578 | 1-methyl-4-phenylpyridinium (MPP) | 200microM | iPSC-derived dopaminergic ne | 24 hours | PE | total RNA | |
| SRP358552 | SRR17898585 | SRR17898579 | 1-methyl-4-phenylpyridinium (MPP) | 200microM | iPSC-derived dopaminergic ne | 24 hours | PE | total RNA | |
| SRP358552 | SRR17898586 | SRR17898580 | 1-methyl-4-phenylpyridinium (MPP) | 200microM | iPSC-derived dopaminergic ne | 24 hours | PE | total RNA | |
| SRP358552 | SRR17898596 | SRR17898590 | 1-methyl-4-phenylpyridinium (MPP) | 200microM | iPSC-derived dopaminergic ne | 24 hours | PE | total RNA | |
| SRP358552 | SRR17898597 | SRR17898591 | 1-methyl-4-phenylpyridinium (MPP) | 200microM | iPSC-derived dopaminergic ne | 24 hours | PE | total RNA | |
| SRP358552 | SRR17898598 | SRR17898592 | 1-methyl-4-phenylpyridinium (MPP) | 200microM | iPSC-derived dopaminergic ne | 24 hours | PE | total RNA | |
| SRP358552 | SRR17898581 | SRR17898578 | 1-methyl-4-phenylpyridinium (MPP) | 400microM | iPSC-derived dopaminergic ne | 24 hours | PE | total RNA | |
| SRP358552 | SRR17898582 | SRR17898579 | 1-methyl-4-phenylpyridinium (MPP) | 400microM | iPSC-derived dopaminergic ne | 24 hours | PE | total RNA | |
| SRP358552 | SRR17898583 | SRR17898580 | 1-methyl-4-phenylpyridinium (MPP) | 400microM | iPSC-derived dopaminergic ne | 24 hours | PE | total RNA | |
| SRP358552 | SRR17898593 | SRR17898590 | 1-methyl-4-phenylpyridinium (MPP) | 400microM | iPSC-derived dopaminergic ne | 24 hours | PE | total RNA | |
| SRP358552 | SRR17898594 | SRR17898591 | 1-methyl-4-phenylpyridinium (MPP) | 400microM | iPSC-derived dopaminergic ne | 24 hours | PE | total RNA | |
| SRP358552 | SRR17898595 | SRR17898592 | 1-methyl-4-phenylpyridinium (MPP) | 400microM | iPSC-derived dopaminergic ne | 24 hours | PE | total RNA | |
| SRP250785 | SRR11184888 | SRR11184889 | radiation | 10Gy | primary astrocytes | Not found | SE | total RNA | |
| SRP250785 | SRR11184890 | SRR11184891 | radiation | 10Gy | primary astrocytes | Not found | SE | total RNA | |
| SRP250785 | SRR11184892 | SRR11184893 | radiation | 10Gy | primary astrocytes | Not found | SE | total RNA | |

*Excel file is available at the figshare repository: https://doi.org/10.6084/m9.figshare.c.7114075.v2

**Supplementary Table2: The result of gene-disease-linker for 168 genes that were differentially expressed in both oxidative stress and Parkinson's disease in human brain.**

| ENSG | ncbi_geneid | GeneSymbol | ONscore | PD_log2fc1 (PMID:37347276) | PD_log2fc2 (PMID: 27611585) | PD_log2fc3 (PMID: 33390883) | TWAS | PD association | Evidence | NU_PMIDs_PD | NU_PMIDs_NCBI | PMIDs_PD | PMIDs_NCBI |
|---|---|---|---|---|---|---|---|---|---|---|---|---|---|
| ENSG00000132437 | 1644 | DDC | -42 | NotFound | -1.5073023 | -2.8602 | NotFound | yes | OpenTargets,DisGeNET,PubChem | 462 | 135 | 19039224, 27 | 1357595, 139 |
| ENSG00000165646 | 6571 | SLC18A2 | 25 | NotFound | -2.3337876 | -3.7445 | NotFound | yes | OpenTargets,DisGeNET,PubChem | 319 | 106 | ppr393667, 3 | 7823026, 790 |
| ENSG00000277586 | 4747 | NEFL | -26 | NotFound | NotFound | -1.3776 | NotFound | yes | OpenTargets,PubChem | 155 | 280 | 36975162, 32 | 1497855, 190 |
| ENSG00000006128 | 6863 | TAC1 | -33 | NotFound | -1.633089 | -1.3407 | NotFound | yes | OpenTargets,PubChem | 151 | 228 | 20582283, 75 | 1312203, 170 |
| ENSG00000170345 | 2353 | FOS | 30 | NotFound | -1.6536481 | NotFound | NotFound | yes | OpenTargets,PubChem | 140 | 520 | 18792992, 21 | 1406655, 154 |
| ENSG00000005381 | 4353 | MPO | 22 | NotFound | 0.42890793 | NotFound | NotFound | yes | OpenTargets,PubChem | 70 | 673 | 24277448, 35 | 1320128, 133 |
| ENSG00000117280 | 8934 | RAB29 | 22 | NotFound | NotFound | NotFound | twas | yes | OpenTargets,PubChem,PubChem | 41 | 59 | 32310270, 19 | 8125298, 928 |
| ENSG00000118785 | 6696 | SPP1 | -35 | NotFound | 0.57900157 | NotFound | NotFound | yes | OpenTargets,PubChem | 33 | 1174 | 17188882, 37 | 1575754, 172 |
| ENSG00000187193 | 4501 | MT1X | 23 | NotFound | 0.40143197 | NotFound | NotFound | yes | OpenTargets,PubChem | 15 | 44 | 18992145, 22 | 2286373, 804 |
| ENSG00000168003 | 6520 | SLC3A2 | 25 | -0.2310392 | NotFound | NotFound | NotFound | yes | PubChem | 15 | 364 | 31277379, 21 | 1358795, 241 |
| ENSG00000169715 | 4493 | MT1E | 38 | 0.63669232 | NotFound | NotFound | NotFound | yes | OpenTargets,PubChem | 14 | 40 | 18992145, 33 | 2286373, 258 |
| ENSG00000205364 | 4499 | MT1M | 32 | NotFound | -1.063394 | NotFound | NotFound | yes | PubChem | 13 | 25 | 33964119, 11 | 2286373, 804 |
| ENSG00000137857 | 53905 | DUOX1 | -21 | 0.47012287 | NotFound | NotFound | NotFound | yes | PubChem | 12 | 82 | 24277523, 21 | 8889548, 106 |
| ENSG00000104722 | 4741 | NEFM | -26 | NotFound | NotFound | -1.1559 | NotFound | yes | OpenTargets,PubChem | 11 | 83 | 32178731, 19 | 1348579, 153 |
| ENSG00000109846 | 1410 | CRYAB | 24 | NotFound | 0.66247107 | 0.3865 | NotFound | yes | OpenTargets | 11 | 387 | 10439447, 25 | 838078, 1407 |
| ENSG00000265972 | 10628 | TXNIP | -24 | NotFound | 0.37324766 | NotFound | NotFound | yes | OpenTargets,PubChem | 11 | 279 | 31939095, 28 | 8086474, 920 |
| ENSG00000145217 | 10861 | SLC26A1 | -23 | NotFound | NotFound | NotFound | twas | yes | OpenTargets | 8 | 19 | 22451204, 22 | 11087667, 11 |
| ENSG00000185294 | 162540 | SPPL2C | 23 | NotFound | NotFound | NotFound | twas | yes | OpenTargets | 7 | 16 | 31701892, 34 | 12139484, 12 |
| ENSG00000146592 | 9586 | CREB5 | 26 | 0.70155554 | NotFound | NotFound | NotFound | yes | OpenTargets | 5 | 64 | 37323142, PI | 8378084, 844 |
| ENSG00000176681 | 9884 | LRRC37A | -31 | NotFound | NotFound | NotFound | twas | yes | OpenTargets | 4 | 11 | 19915575, 22 | 9628581, 124 |
| ENSG00000103257 | 8140 | SLC7A5 | 24 | -0.336146 | NotFound | NotFound | NotFound | yes | OpenTargets | 4 | 298 | 20606323, 36 | 1597461, 756 |
| ENSG00000242808 | 347689 | SOX2-OT | -25 | 0.68639499 | -1.2890891 | NotFound | NotFound | yes | OpenTargets,RNAdisease | 3 | 60 | 34198485, 27 | 12477932, 12 |
| ENSG00000129757 | 1028 | CDKN1C | -21 | 0.77925983 | 0.59859045 | 0.5561 | NotFound | yes | OpenTargets | 3 | 248 | ppr393667, 3 | 1786629, 772 |
| ENSG00000085514 | 29992 | PILRA | 40 | NotFound | NotFound | NotFound | twas | yes | OpenTargets | 2 | 34 | 34206597, 34 | 10660620, 10 |
| ENSG00000110090 | 1374 | CPT1A | 22 | -0.2147354 | NotFound | NotFound | NotFound | yes | OpenTargets | 2 | 201 | 32973137, 32 | 7892212, 907 |
| ENSG00000019582 | 972 | CD74 | 22 | NotFound | 0.46894416 | NotFound | NotFound | yes | OpenTargets | 2 | 209 | 36926730, 36 | 1448172, 212 |
| ENSG00000197558 | | SSPOP | 24 | NotFound | -1.5713875 | NotFound | NotFound | yes | OpenTargets | 2 | 0 | 32099673, 33946285 | |
| ENSG00000167191 | 51704 | GPRC5B | -34 | NotFound | 0.51303902 | NotFound | NotFound | yes | OpenTargets | 1 | 44 | 24391664 | 10493829, 10 |
| ENSG00000158352 | 57477 | SHROOM4 | 23 | 0.53742204 | NotFound | NotFound | NotFound | yes | OpenTargets | 1 | 15 | ppr393667 | 10574462, 10 |
| ENSG00000104833 | 10382 | TUBB4A | -30 | NotFound | 0.37894422 | NotFound | NotFound | yes | OpenTargets | 1 | 207 | 28171541 | 3782288, 646 |
| ENSG00000162551 | 249 | ALPL | -23 | -0.5748171 | NotFound | NotFound | NotFound | yes | OpenTargets | 1 | 297 | 37165152 | 1321014, 140 |
| ENSG00000095397 | 25861 | WHRN | 24 | 0.62860282 | 0.41330998 | NotFound | NotFound | yes | OpenTargets | 1 | 41 | 34680087 | 10819331, 11 |
| ENSG00000043143 | 23338 | JADE2 | 29 | NotFound | 0.38796059 | NotFound | NotFound | yes | OpenTargets | 1 | 33 | 31701892 | 9039502, 124 |
| ENSG00000225151 | | GOLGA2P7 | 25 | -0.3109294 | NotFound | NotFound | NotFound | yes | OpenTargets | 1 | 0 | 33417599 | |
| ENSG00000107731 | 219699 | UNC5B | 21 | NotFound | 0.38018109 | NotFound | NotFound | yes | OpenTargets | 1 | 76 | 37039476 | 12107411, 12 |
| ENSG00000115041 | 30818 | KCNIP3 | 32 | NotFound | NotFound | NotFound | twas | yes | OpenTargets | 1 | 81 | 31701892 | 9771752, 981 |
| ENSG00000258947 | 10381 | TUBB3 | -21 | NotFound | -0.809495 | -0.8964 | NotFound | yes | OpenTargets | 1 | 352 | 36206343 | 2461292, 345 |
| ENSG00000249859 | | PVT1 | 30 | -0.2891514 | NotFound | NotFound | NotFound | yes | OpenTargets | 1 | 0 | 35978647 | |
| ENSG00000198821 | 919 | CD247 | 29 | NotFound | -1.2065512 | NotFound | NotFound | yes | OpenTargets | 1 | 262 | 20972807 | 1385158, 138 |
| ENSG00000080845 | 22839 | DLGAP4 | 21 | -0.2166951 | NotFound | NotFound | NotFound | yes | OpenTargets | 1 | 40 | 37186176 | 9115257, 102 |
| ENSG00000263934 | | SNORD3A | -20 | -1.576952 | NotFound | NotFound | NotFound | yes | OpenTargets | 1 | 0 | 23349890 | |
| ENSG00000170075 | 9283 | GPR37L1 | 42 | -0.3087951 | NotFound | NotFound | NotFound | yes | OpenTargets | 1 | 23 | 33208571 | 8125298, 953 |
| ENSG00000185022 | 23764 | MAFF | 23 | NotFound | -0.8587779 | 1.4136 | NotFound | yes | OpenTargets | 1 | 56 | 33488846 | 8107826, 881 |
| ENSG00000107281 | 56654 | NPDC1 | 28 | -0.2169371 | NotFound | NotFound | NotFound | yes | OpenTargets | 1 | 25 | 30025813 | 7878019, 812 |
| ENSG00000109610 | 6649 | SOD3 | -25 | NotFound | -0.979237 | NotFound | NotFound | yes | OpenTargets | 1 | 218 | 31601079 | 1477980, 150 |
| ENSG00000084453 | 6579 | SLCO1A2 | 33 | 0.71833779 | NotFound | NotFound | NotFound | yes | OpenTargets | 1 | 66 | 22685416 | 7557095, 900 |
| ENSG00000023330 | 211 | ALAS1 | 24 | NotFound | NotFound | -0.4073 | NotFound | yes | OpenTargets | 1 | 75 | 34885088 | 2095458, 226 |
| ENSG00000276168 | 6029 | RN7SL1 | -25 | -0.2472332 | NotFound | NotFound | NotFound | yes | RNAdisease | 1 | 12 | 27021022 | 6084597, 120 |
| ENSG00000173110 | 3310 | HSPA6 | 50 | NotFound | 1.58868146 | NotFound | NotFound | yes | OpenTargets | 1 | 198 | 36104441 | 1346391, 232 |
| ENSG00000112333 | 7101 | NR2E1 | -21 | NotFound | 1.53613548 | NotFound | NotFound | yes | OpenTargets | 1 | 60 | ppr393667 | 2570372, 804 |
| ENSG00000163288 | 2560 | GABRB1 | -30 | NotFound | -0.9304768 | NotFound | NotFound | yes | OpenTargets | 1 | 58 | ppr393667 | 1321150, 132 |
| ENSG00000180739 | 53637 | S1PR5 | 24 | 0.96567904 | NotFound | NotFound | NotFound | no | - | 0 | 41 | - | 2834384, 107 |
| ENSG00000230487 | 114796 | PSMG3-AS1 | -21 | -0.3816003 | -4.249603 | NotFound | NotFound | no | - | 0 | 12 | - | 11572484, 12 |
| ENSG00000178826 | 135932 | TMEM139 | 22 | 1.15569124 | NotFound | NotFound | NotFound | no | - | 0 | 18 | - | 8889548, 124 |
| ENSG00000176046 | 26471 | NUPR1 | 23 | NotFound | 0.72807831 | 0.6137 | twas | no | - | 0 | 101 | - | 9405444, 100 |
| ENSG00000008710 | 5310 | PKD1 | 22 | NotFound | NotFound | 0.3899 | NotFound | no | - | 0 | 354 | - | 1577479, 282 |
| ENSG00000080573 | 50509 | COL5A3 | -30 | -0.5891189 | NotFound | 0.4952 | NotFound | no | - | 0 | 25 | - | 1571108, 191 |
| ENSG00000182263 | 55137 | FIGN | -20 | NotFound | 0.43903049 | NotFound | NotFound | no | - | 0 | 26 | - | 11017077, 14 |
| ENSG00000258725 | 100507118 | PRC1-AS1 | 40 | -0.5162268 | NotFound | NotFound | NotFound | no | - | 0 | 4 | - | 16344560, 20 |
| ENSG00000164483 | 154075 | SAMD3 | 26 | NotFound | 1.27638715 | NotFound | NotFound | no | - | 0 | 15 | - | 12477932, 14 |
| ENSG00000237943 | 439949 | PRKCQ-AS1 | 30 | 0.52310192 | NotFound | NotFound | NotFound | no | - | 0 | 6 | - | 8619474, 911 |
| ENSG00000214900 | 196913 | LINC01588 | 42 | NotFound | 1.10270573 | NotFound | NotFound | no | - | 0 | 4 | - | 12477932, 16 |
| ENSG00000106278 | 5803 | PTPRZ1 | -23 | NotFound | -0.7305248 | NotFound | NotFound | no | - | 0 | 95 | - | 1323835, 216 |
| ENSG00000204361 | 120406 | NXPE2 | 21 | NotFound | -1.9414343 | NotFound | NotFound | no | - | 0 | 4 | - | 12477932, 24 |
| ENSG00000228789 | 285834 | HCG22 | -21 | NotFound | -1.7811092 | NotFound | NotFound | no | - | 0 | 14 | - | 14702039, 19 |
| ENSG00000164124 | 55314 | TMEM144 | 21 | 0.91236023 | -0.8296603 | NotFound | NotFound | no | - | 0 | 8 | - | 12477932, 14 |
| ENSG00000224086 | | | -24 | -0.5437507 | NotFound | NotFound | NotFound | no | - | 0 | 0 | - | |
| ENSG00000280160 | | | -20 | 0.45314671 | NotFound | NotFound | NotFound | no | - | 0 | 0 | - | |
| ENSG00000147996 | 220869 | ZNG1E | 21 | NotFound | -0.7409363 | NotFound | NotFound | no | - | 0 | 19 | - | 8889548, 888 |
| ENSG00000260947 | | | -20 | 0.40345302 | NotFound | NotFound | NotFound | no | - | 0 | 0 | - | |
| ENSG00000122584 | 30010 | NXPH1 | -24 | NotFound | -0.7396528 | NotFound | NotFound | no | - | 0 | 32 | - | 8699246, 957 |
| ENSG00000167077 | 150365 | MEI1 | 24 | NotFound | NotFound | NotFound | twas | no | - | 0 | 13 | - | 12477932, 14 |
| ENSG00000127804 | 79066 | METTL16 | 21 | NotFound | 0.40940264 | NotFound | NotFound | no | - | 0 | 66 | - | 7698749, 124 |
| ENSG00000168743 | 255743 | NPNT | -33 | NotFound | -1.1649453 | NotFound | NotFound | no | - | 0 | 30 | - | 8889548, 114 |
| ENSG00000138050 | 80745 | THUMPD2 | 22 | 0.27514223 | NotFound | NotFound | NotFound | no | - | 0 | 16 | - | 8125298, 120 |
| ENSG00000141668 | 147381 | CBLN2 | -21 | NotFound | -1.2498725 | NotFound | NotFound | no | - | 0 | 13 | - | 7877445, 124 |
| ENSG00000162616 | 11080 | DNAJB4 | 23 | NotFound | 0.51016861 | NotFound | NotFound | no | - | 0 | 89 | - | 8889548, 954 |
| ENSG00000153714 | 286343 | LURAP1L | 35 | 0.46894001 | NotFound | NotFound | NotFound | no | - | 0 | 17 | - | 12477932, 12 |
| ENSG00000138311 | 22891 | ZNF365 | 21 | NotFound | -1.0728972 | NotFound | NotFound | no | - | 0 | 55 | - | 10048485, 11 |
| ENSG00000138650 | 57575 | PCDH10 | -22 | NotFound | -0.8464891 | NotFound | NotFound | no | - | 0 | 72 | - | 8012384, 103 |
| ENSG00000146151 | 54511 | HMGCLL1 | -20 | NotFound | -1.1131504 | NotFound | NotFound | no | - | 0 | 15 | - | 8440722, 861 |
| ENSG00000020577 | 23034 | SAMD4A | 33 | NotFound | 0.38502149 | NotFound | NotFound | no | - | 0 | 37 | - | 10470851, 12 |
| ENSG00000136059 | 50853 | VILL | -26 | -0.6128217 | NotFound | NotFound | NotFound | no | - | 0 | 12 | - | 9179494, 124 |
| ENSG00000130702 | 3911 | LAMA5 | 24 | -0.4649711 | NotFound | NotFound | NotFound | no | - | 0 | 159 | - | 7499364, 900 |
| ENSG00000233760 | | | -29 | NotFound | -1.2426877 | NotFound | NotFound | no | - | 0 | 0 | - | |
| ENSG00000103932 | 26015 | RPAP1 | 24 | NotFound | NotFound | NotFound | twas | no | - | 0 | 53 | - | 10718198, 12 |
| ENSG00000056736 | 55540 | IL17RB | -20 | NotFound | NotFound | 0.5091 | NotFound | no | - | 0 | 64 | - | 10748240, 10 |
| ENSG00000179954 | 284297 | SSC5D | 34 | NotFound | 1.62329274 | NotFound | NotFound | no | - | 0 | 10 | - | 12477932, 14 |
| ENSG00000147854 | 115426 | UHRF2 | 25 | 0.28755659 | 0.57514067 | NotFound | NotFound | no | - | 0 | 71 | - | 12176013, 12 |
| ENSG00000286558 | | | 28 | 1.18618066 | NotFound | NotFound | NotFound | no | - | 0 | 0 | - | |

| | | | | | | | | | | | | | |
|---|---|---|---|---|---|---|---|---|---|---|---|---|---|
| ENSG00000196436 | 124907807 | NPIPB15 | 35 | NotFound | 1.29234343 | NotFound | NotFound | no | - | 0 | 0 | - | |
| ENSG00000290926 | 441242 | LINC03006 | 28 | NotFound | -2.1114183 | NotFound | NotFound | no | - | 0 | 3 | - | 12477932, 14 |
| ENSG00000176571 | 168975 | CNBD1 | -42 | NotFound | 1.26235108 | NotFound | NotFound | no | - | 0 | 6 | - | 12477932, 14 |
| ENSG00000215595 | 400831 | C20orf202 | 23 | NotFound | -1.0314603 | NotFound | NotFound | no | - | 0 | 3 | - | 12477932, 14 |
| ENSG00000169129 | 84632 | AFAP1L2 | -24 | -0.2461164 | NotFound | NotFound | NotFound | no | - | 0 | 47 | - | 10737800, 11 |
| ENSG00000125703 | 84938 | ATG4C | 27 | 0.28151935 | 0.58779044 | NotFound | NotFound | no | - | 0 | 30 | - | 8889548, 124 |
| ENSG00000170509 | 345275 | HSD17B13 | 23 | NotFound | -1.1621997 | NotFound | NotFound | no | - | 0 | 48 | - | 12477932, 12 |
| ENSG00000197705 | 57565 | KLHL14 | -36 | NotFound | -1.6648023 | NotFound | NotFound | no | - | 0 | 14 | - | 10718198, 12 |
| ENSG00000244491 | | | -33 | 1.00668112 | NotFound | NotFound | NotFound | no | - | 0 | 0 | - | |
| ENSG00000204241 | 100128239 | LINC02731 | 27 | -0.663261 | NotFound | NotFound | NotFound | no | - | 0 | 1 | - | 14702039 |
| ENSG00000170537 | 79905 | TMC7 | 25 | 0.62731938 | NotFound | NotFound | NotFound | no | - | 0 | 13 | - | 12477932, 12 |
| ENSG00000087116 | 9509 | ADAMTS2 | 38 | NotFound | -1.7377827 | NotFound | NotFound | no | - | 0 | 39 | - | 7735500, 100 |
| ENSG00000145794 | 84466 | MEGF10 | -44 | NotFound | 0.4545622 | NotFound | NotFound | no | - | 0 | 37 | - | 11347906, 12 |
| ENSG00000287299 | | | 34 | 0.59436733 | NotFound | NotFound | NotFound | no | - | 0 | 0 | - | |
| ENSG00000175093 | 92369 | SPSB4 | -23 | NotFound | -1.5916647 | NotFound | NotFound | no | - | 0 | 22 | - | 12076535, 12 |
| ENSG00000101977 | 4168 | MCF2 | 29 | NotFound | NotFound | -0.6875 | NotFound | no | - | 0 | 39 | - | 1611909, 206 |
| ENSG00000229337 | | | 36 | 0.85557577 | NotFound | NotFound | NotFound | no | - | 0 | 0 | - | |
| ENSG00000197815 | | | -23 | 0.51911267 | NotFound | NotFound | NotFound | no | - | 0 | 0 | - | |
| ENSG00000143110 | 128346 | C1orf162 | 28 | NotFound | 0.40981582 | NotFound | NotFound | no | - | 0 | 7 | - | 7566098, 124 |
| ENSG00000197457 | 50861 | STMN3 | -23 | -0.2321685 | NotFound | NotFound | NotFound | no | - | 0 | 33 | - | 9603203, 978 |
| ENSG00000066382 | 744 | MPPED2 | -27 | NotFound | NotFound | -1.1341 | NotFound | no | - | 0 | 30 | - | 7527372, 866 |
| ENSG00000186265 | 151888 | BTLA | 39 | NotFound | 1.12182686 | NotFound | NotFound | no | - | 0 | 91 | - | 10737800, 12 |
| ENSG00000137648 | 56649 | TMPRSS4 | 38 | NotFound | NotFound | -0.7376 | NotFound | no | - | 0 | 65 | - | 8889548, 108 |
| ENSG00000184545 | 1850 | DUSP8 | 23 | NotFound | NotFound | NotFound | twas | no | - | 0 | 22 | - | 7561881, 888 |
| ENSG00000265763 | 118738 | ZNF488 | -23 | NotFound | 0.48366976 | NotFound | NotFound | no | - | 0 | 15 | - | 12477932, 14 |
| ENSG00000184515 | 340542 | BEX5 | -22 | NotFound | -1.665453 | NotFound | NotFound | no | - | 0 | 16 | - | 12477932, 15 |
| ENSG00000136698 | 55997 | CFC1 | 23 | NotFound | -0.7619324 | NotFound | NotFound | no | - | 0 | 25 | - | 7747776, 108 |
| ENSG00000233723 | | | -33 | NotFound | -1.149437 | NotFound | NotFound | no | - | 0 | 0 | - | |
| ENSG00000005249 | 5577 | PRKAR2B | -21 | NotFound | NotFound | -1.4002 | NotFound | no | - | 0 | 133 | - | 1332964, 135 |
| ENSG00000140600 | 6457 | SH3GL3 | -20 | 0.30584681 | NotFound | NotFound | NotFound | no | - | 0 | 61 | - | 9122235, 916 |
| ENSG00000177875 | 387856 | CCDC184 | -23 | NotFound | -0.985532 | NotFound | NotFound | no | - | 0 | 13 | - | 11076863, 11 |
| ENSG00000129673 | 15 | AANAT | 31 | NotFound | -1.9407304 | NotFound | NotFound | no | - | 0 | 45 | - | 2181999, 750 |
| ENSG00000228824 | 642345 | MIR4500HG | -27 | NotFound | -1.1187478 | NotFound | NotFound | no | - | 0 | 4 | - | 11181995, 11 |
| ENSG00000196187 | 9725 | TMEM63A | 21 | 0.8462386 | NotFound | NotFound | NotFound | no | - | 0 | 28 | - | 8125298, 945 |
| ENSG00000185681 | 254956 | MORN5 | -24 | NotFound | -1.0042876 | NotFound | NotFound | no | - | 0 | 6 | - | 12477932, 20 |
| ENSG00000284505 | 111188157 | LYNX1-SLUF | 23 | -0.3476224 | NotFound | NotFound | NotFound | no | - | 0 | 3 | - | 11181995, 12 |
| ENSG00000248801 | | | -23 | NotFound | -2.489349 | NotFound | NotFound | no | - | 0 | 0 | - | |
| ENSG00000166888 | 6778 | STAT6 | 21 | -0.2381958 | NotFound | NotFound | NotFound | no | - | 0 | 393 | - | 7694370, 776 |
| ENSG00000101251 | 80343 | SEL1L2 | 28 | NotFound | -0.8792312 | NotFound | NotFound | no | - | 0 | 8 | - | 12477932, 14 |
| ENSG00000099769 | 3483 | IGFALS | 27 | NotFound | NotFound | NotFound | twas | no | - | 0 | 67 | - | 1379671, 138 |
| ENSG00000132205 | 84034 | EMILIN2 | 24 | NotFound | -1.1085089 | NotFound | NotFound | no | - | 0 | 32 | - | 11278945, 12 |
| ENSG00000112893 | 4124 | MAN2A1 | 26 | 0.48796667 | 0.57502653 | NotFound | NotFound | no | - | 0 | 72 | - | 1736542, 175 |
| ENSG00000115884 | 6382 | SDC1 | 26 | NotFound | -0.9650419 | NotFound | NotFound | no | - | 0 | 429 | - | 1339431, 144 |
| ENSG00000249715 | 90342 | FER1L5 | 26 | NotFound | 1.1332181 | NotFound | NotFound | no | - | 0 | 8 | - | 11181995, 12 |
| ENSG00000157551 | 3772 | KCNJ15 | 25 | 0.35869934 | NotFound | NotFound | NotFound | no | - | 0 | 33 | - | 8995301, 903 |
| ENSG00000114541 | 23150 | FRMD4B | -20 | 0.63632727 | NotFound | NotFound | NotFound | no | - | 0 | 20 | - | 10231032, 11 |
| ENSG00000226609 | | | 23 | 0.86485942 | NotFound | NotFound | NotFound | no | - | 0 | 0 | - | |
| ENSG00000126860 | 2123 | EVI2A | 24 | 0.88685387 | NotFound | NotFound | NotFound | no | - | 0 | 23 | - | 1639383, 171 |
| ENSG00000267454 | 386758 | ZNF582-DT | 23 | NotFound | NotFound | NotFound | twas | no | - | 0 | 1 | - | 12477932 |
| ENSG00000179796 | 116135 | LRRC3B | -23 | NotFound | -0.8302146 | NotFound | NotFound | no | - | 0 | 21 | - | 11076863, 12 |
| ENSG00000278963 | | | 24 | 0.42859113 | NotFound | NotFound | NotFound | no | - | 0 | 0 | - | |
| ENSG00000004799 | 5166 | PDK4 | 24 | NotFound | 0.91436918 | NotFound | NotFound | no | - | 0 | 103 | - | 7499431, 812 |
| ENSG00000273015 | 400027 | LINC00938 | -20 | 0.26620488 | NotFound | NotFound | NotFound | yes | RNAdisease | 1 | 0 | 35173238 | |
| ENSG00000224738 | | | 26 | 0.44089344 | NotFound | NotFound | NotFound | no | - | 0 | 0 | - | |
| ENSG00000178038 | 259173 | ALS2CL | 24 | -0.4954791 | NotFound | NotFound | NotFound | no | - | 0 | 12 | - | 8889548, 124 |
| ENSG00000286480 | | | -21 | 0.61540172 | NotFound | NotFound | NotFound | no | - | 0 | 0 | - | |
| ENSG00000186377 | 260293 | CYP4X1 | -37 | NotFound | -0.823365 | NotFound | NotFound | no | - | 0 | 17 | - | 12176035, 12 |
| ENSG00000139629 | 11226 | GALNT6 | 34 | 0.86406822 | NotFound | NotFound | NotFound | no | - | 0 | 40 | - | 8125298, 104 |
| ENSG00000070882 | 26031 | OSBPL3 | 27 | NotFound | -0.9731236 | NotFound | NotFound | no | - | 0 | 77 | - | 9734811, 984 |
| ENSG00000100341 | 150379 | PNPLA5 | -22 | NotFound | -1.3101791 | NotFound | NotFound | no | - | 0 | 14 | - | 12477932, 14 |
| ENSG00000144908 | 10840 | ALDH1L1 | 21 | -0.4388865 | NotFound | NotFound | NotFound | no | - | 0 | 79 | - | 2733692, 797 |
| ENSG00000112796 | 59084 | ENPP5 | 25 | NotFound | -0.7208764 | NotFound | NotFound | no | - | 0 | 13 | - | 11027689, 12 |
| ENSG00000205837 | 400941 | LINC00487 | -20 | NotFound | 1.12813703 | NotFound | NotFound | no | - | 0 | 0 | - | |
| ENSG00000130822 | 139728 | PNCK | 31 | -0.3485787 | NotFound | NotFound | NotFound | no | - | 0 | 13 | - | 8889548, 106 |
| ENSG00000203814 | 440689 | H2BC18 | -20 | NotFound | -1.2750109 | NotFound | NotFound | no | - | 0 | 59 | - | 12408966, 12 |
| ENSG00000117525 | 2152 | F3 | 40 | NotFound | 0.41610083 | NotFound | NotFound | no | - | 0 | 644 | - | 392457, 1740 |
| ENSG00000162592 | 148870 | CCDC27 | 21 | NotFound | 1.09294892 | NotFound | NotFound | no | - | 0 | 17 | - | 12477932, 14 |
| ENSG00000113645 | 23286 | WWC1 | 25 | -0.2005931 | NotFound | NotFound | NotFound | no | - | 0 | 138 | - | 8889548, 100 |
| ENSG00000274266 | | | -27 | -1.0246473 | NotFound | NotFound | NotFound | no | - | 0 | 0 | - | |
| ENSG00000078596 | 9452 | ITM2A | 21 | NotFound | -0.8017252 | NotFound | NotFound | no | - | 0 | 36 | - | 8125298, 857 |
| ENSG00000091137 | 5172 | SLC26A4 | 21 | NotFound | -0.8075217 | NotFound | NotFound | no | - | 0 | 322 | - | 8541853, 863 |
| ENSG00000124092 | 140690 | CTCFL | 28 | NotFound | 0.39220138 | NotFound | NotFound | no | - | 0 | 91 | - | 11780052, 12 |
| ENSG00000274020 | | | -27 | 0.2459816 | NotFound | NotFound | NotFound | no | - | 0 | 0 | - | |
| ENSG00000243836 | 100131176 | WDR86-AS1 | -22 | NotFound | -1.2625438 | NotFound | NotFound | no | - | 0 | 3 | - | 14702039, 16 |
| ENSG00000137955 | 5876 | RABGGTB | 23 | 0.17012087 | NotFound | NotFound | NotFound | no | - | 0 | 56 | - | 546546, 1598 |
| ENSG00000198286 | 84433 | CARD11 | 27 | NotFound | 1.13576928 | NotFound | NotFound | no | - | 0 | 136 | - | 8889549, 984 |
| ENSG00000181544 | 2187 | FANCB | 25 | 0.35828322 | NotFound | NotFound | NotFound | no | - | 0 | 50 | - | 8609606, 938 |
| ENSG00000258791 | 645687 | LINC00520 | -28 | NotFound | 3.31830951 | NotFound | NotFound | no | - | 0 | 16 | - | 23251661, 25 |

The complete tsv file is available from the figshare repository: https://doi.org/10.6084/m9.figshare.c.7114075.v2

**Supplementary Table3: The reference file used to convert transcript IDs to gene IDs using tximport.**

| | |
|---|---|
| ENST00000387314.1 | ENSG00000210049 |
| ENST00000389680.2 | ENSG00000211459 |
| ENST00000387342.1 | ENSG00000210077 |
| ENST00000387347.2 | ENSG00000210082 |
| ENST00000386347.1 | ENSG00000209082 |
| ENST00000361390.2 | ENSG00000198888 |
| ENST00000387365.1 | ENSG00000210100 |
| ENST00000387372.1 | ENSG00000210107 |
| ENST00000387377.1 | ENSG00000210112 |
| ENST00000361453.3 | ENSG00000198763 |
| ENST00000387382.1 | ENSG00000210117 |
| ENST00000387392.1 | ENSG00000210127 |
| ENST00000387400.1 | ENSG00000210135 |
| ENST00000387405.1 | ENSG00000210140 |
| ENST00000387409.1 | ENSG00000210144 |
| ENST00000361624.2 | ENSG00000198804 |
| ENST00000387416.2 | ENSG00000210151 |
| ENST00000387419.1 | ENSG00000210154 |
| ENST00000361739.1 | ENSG00000198712 |
| ENST00000387421.1 | ENSG00000210156 |
| ENST00000361851.1 | ENSG00000228253 |
| ENST00000361899.2 | ENSG00000198899 |
| ENST00000362079.2 | ENSG00000198938 |
| ENST00000387429.1 | ENSG00000210164 |
| ENST00000361227.2 | ENSG00000198840 |
| ENST00000387439.1 | ENSG00000210174 |
| ENST00000361335.1 | ENSG00000212907 |
| ENST00000361381.2 | ENSG00000198886 |
| ENST00000387441.1 | ENSG00000210176 |
| ENST00000387449.1 | ENSG00000210184 |
| ENST00000387456.1 | ENSG00000210191 |
| ENST00000361567.2 | ENSG00000198786 |
| ENST00000361681.2 | ENSG00000198695 |
| ENST00000387459.1 | ENSG00000210194 |
| ENST00000361789.2 | ENSG00000198727 |
| ENST00000387460.2 | ENSG00000210195 |
| ENST00000387461.2 | ENSG00000210196 |
| ENST00000620265.1 | ENSG00000278457 |
| ENST00000619317.1 | ENSG00000276197 |
| ENST00000616830.1 | ENSG00000278625 |
| ENST00000614336.4 | ENSG00000271254 |
| ENST00000612640.4 | ENSG00000271254 |
| ENST00000619792.1 | ENSG00000278633 |
| ENST00000616049.4 | ENSG00000277630 |

The complete file is available from the figshare repository: https://doi.org/10.6084/m9.figshare.c.7114075.v2

**Supplementary Table4: The result of DESeq2 analysis in oxidative stress RNA-seq datasets.**

| | baseMean | log2FoldCha | lfcSE | stat | pvalue | padj |
|---|---|---|---|---|---|---|
| ENSG00000251730 | 13.9135116 | 24.4063135 | 1.49567042 | 16.3179757 | 7.35E-60 | 1.67E-55 |
| ENSG00000285794 | 19.4634929 | 24.9034832 | 1.66986415 | 14.9134786 | 2.69E-50 | 3.05E-46 |
| ENSG00000206609 | 9.82616825 | 23.937175 | 1.66279284 | 14.395765 | 5.50E-47 | 4.16E-43 |
| ENSG00000252699 | 22.5884802 | 25.093734 | 1.81293927 | 13.8414642 | 1.43E-43 | 8.12E-40 |
| ENSG00000276103 | 20.7324139 | 24.9807639 | 1.85003829 | 13.5028362 | 1.50E-41 | 5.69E-38 |
| ENSG00000285522 | 24.0699985 | 25.1822784 | 1.86447193 | 13.5063865 | 1.43E-41 | 5.69E-38 |
| ENSG00000207975 | 7.61032212 | 23.5281705 | 2.14112845 | 10.9886777 | 4.33E-28 | 1.40E-24 |
| ENSG00000266307 | 6.20477319 | 23.3060395 | 2.12430544 | 10.9711339 | 5.26E-28 | 1.49E-24 |
| ENSG00000201882 | 30.516912 | 25.5314658 | 2.56347442 | 9.95971157 | 2.29E-23 | 5.25E-20 |
| ENSG00000222345 | 27.9808418 | 25.4035701 | 2.55094833 | 9.95848084 | 2.32E-23 | 5.25E-20 |
| ENSG00000252542 | 34.4696288 | 25.4980108 | 2.57934463 | 9.88546101 | 4.81E-23 | 9.92E-20 |
| ENSG00000277941 | 16.1015332 | 23.3699959 | 2.46698897 | 9.47308487 | 2.72E-21 | 5.13E-18 |
| ENSG00000050767 | 70.7271916 | -3.0060479 | 0.31803186 | -9.4520338 | 3.32E-21 | 5.80E-18 |
| ENSG00000065618 | 48.5524917 | -3.179097 | 0.36689842 | -8.664788 | 4.52E-18 | 7.33E-15 |
| ENSG00000284425 | 22.8181757 | 25.122268 | 3.17708645 | 7.90732905 | 2.63E-15 | 3.97E-12 |
| ENSG00000182912 | 97.9325309 | -3.2849394 | 0.42671233 | -7.6982528 | 1.38E-14 | 1.84E-11 |
| ENSG00000266852 | 14.2773015 | 24.4664832 | 3.17716001 | 7.70074001 | 1.35E-14 | 1.84E-11 |
| ENSG00000276758 | 13.147171 | 24.3550425 | 3.17717692 | 7.66562365 | 1.78E-14 | 2.24E-11 |
| ENSG00000278267 | 12.641417 | 24.293015 | 3.17718544 | 7.64608031 | 2.07E-14 | 2.47E-11 |
| ENSG00000275134 | 9.49558791 | 23.8987704 | 3.1772589 | 7.52182027 | 5.40E-14 | 6.12E-11 |
| ENSG00000160307 | 42.5279389 | -1.4573097 | 0.221813 | -6.5699921 | 5.03E-11 | 5.43E-08 |
| ENSG00000228709 | 43.5393396 | -2.5787112 | 0.39855652 | -6.4701267 | 9.79E-11 | 1.01E-07 |
| ENSG00000112715 | 742.316879 | -1.4785192 | 0.24105514 | -6.1335308 | 8.59E-10 | 8.47E-07 |
| ENSG00000138081 | 611.651281 | -0.255538 | 0.04226908 | -6.0455059 | 1.49E-09 | 1.41E-06 |
| ENSG00000253764 | 32.0672171 | -1.6376603 | 0.27174943 | -6.0263615 | 1.68E-09 | 1.52E-06 |
| ENSG00000136160 | 53.8576385 | -1.9555037 | 0.3254122 | -6.0093127 | 1.86E-09 | 1.62E-06 |
| ENSG00000235890 | 121.51574 | -3.4841487 | 0.58090721 | -5.9977716 | 2.00E-09 | 1.68E-06 |
| ENSG00000231389 | 36.0035862 | -2.5849823 | 0.44577103 | -5.7989015 | 6.68E-09 | 5.40E-06 |
| ENSG00000169136 | 377.301978 | 0.61254215 | 0.10590524 | 5.78387001 | 7.30E-09 | 5.71E-06 |
| ENSG00000175147 | 19.7760819 | -2.3736361 | 0.4175162 | -5.6851353 | 1.31E-08 | 9.88E-06 |
| ENSG00000073060 | 369.189002 | -1.1735849 | 0.20774624 | -5.6491269 | 1.61E-08 | 1.18E-05 |
| ENSG00000100453 | 7.18198755 | 3.00880443 | 0.56761675 | 5.30076748 | 1.15E-07 | 7.69E-05 |
| ENSG00000160336 | 149.066697 | 0.34473475 | 0.06497159 | 5.30593078 | 1.12E-07 | 7.69E-05 |
| ENSG00000227617 | 16.3716695 | -3.2496831 | 0.61183386 | -5.3113816 | 1.09E-07 | 7.69E-05 |
| ENSG00000105894 | 1704.49103 | -1.9380231 | 0.3663466 | -5.2901352 | 1.22E-07 | 7.92E-05 |
| ENSG00000080166 | 331.809416 | 2.47507369 | 0.47186305 | 5.24532211 | 1.56E-07 | 9.82E-05 |
| ENSG00000006611 | 11.5181896 | 2.0761043 | 0.40128737 | 5.17360985 | 2.30E-07 | 0.00013339 |
| ENSG00000100292 | 426.576587 | 1.12799214 | 0.21822276 | 5.1689941 | 2.35E-07 | 0.00013339 |
| ENSG00000105650 | 49.0369225 | -1.779487 | 0.34401239 | -5.1727412 | 2.31E-07 | 0.00013339 |

The complete excel file is available from the figshare repository: https://doi.org/10.6084/m9.figshare.c.7114075.v2

**Supplementary Data5: The script used for conducting Fisher's exact test to determine the statistical correlation between the gene sets obtained by meta-analysis and DESeq2.**

In [ ]:

```
import numpy as np
from scipy.stats import fisher_exact

# set data
both_ON_deseq = 89
only_ON = 3025
only_deseq = 263
neither_ON_deseq = 58889

observed = np.array([
    [both_ON_deseq, only_ON],
    [only_deseq, neither_ON_deseq]
])

# fisher exact test
odds_ratio, p_value = fisher_exact(observed)

# results
print(f"odds ratio: {odds_ratio}")
print(f"p-value: {p_value}")


# interpret the results
if p_value < 0.05:
    print(
        """At a significance level of 5%,
          there is a statistically significant correlation between genes
          by meta-analysis method and genes by DESeq2."""
    )
else:
    print(
        """At a significance level of 5%,
        there is NO statistically significant correlation between genes
        by meta-analysis method and genes by DESeq2."""
    )
```

In [ ]:

```
# The python file is available from figshare repository: https://doi.org/10.6084/m9.figshare.c.7114075.v2
```

The python file is available from the figshare repository: https://doi.org/10.6084/m9.figshare.c.7114075.v2
